# Supplementary material for: RNA fine-tunes estrogen receptor-alpha binding on low-affinity DNA motifs for transcriptional regulation
Source: EMBO J. 2024 Sep 16;43(21):5186–210. doi: 10.1038/s44318-024-00225-y (PMC11535219; doi:10.1038/s44318-024-00225-y)
Supplement: Supplementary file 1 — Appendix [file 44318_2024_225_MOESM1_ESM.pdf]

RNA fine-tunes estrogen receptor-alpha binding on low-affinity DNA motifs for transcriptional regulation

Deepanshu Soota[1], Bharath Saravanan[1, 2], Rajat Mann[1], Tripti Kharbanda[1], Dimple Notani[1#]

Affiliations:

1. National Center for Biological Sciences, Tata Institute for Fundamental Research, Bangalore, Karnataka, 560065, India

2. Sastra Deemed University, Thanjavur, Tamil Nadu, 613401. India

# Corresponding Author

Appendix Table of Contents

Appendix Table S1……………………………………………………………………………………2

Appendix Table S2……………………………………………………………………………………2

Appendix Table S3……………………………………………………………………………………2

Appendix Table S4…………………………………………………………………………………2-3

Appendix Table S5……………………………………………………………………………………3

Appendix Table S6…………………………………………………………………………………3-5

Appendix Table S1: Primers used for cloning

TFF1eRNA cloning	Forward Primer	Reverse Primer
Fragment 1	5'GATAAGCTTGTTTTCAGCACAAGCGTCTG3'	5'GATCTCGAGATTCCTGGGCTTGTAGCTGG3'
Fragment 2	5'GATAAGCTTGCCAGTGCAGCTGCAGCCGC3'	5'GATCTCGAGCCAACAATAATTTTAGAGCC3'
Fragment 3	5'GATGAATTCAACTACAGCCCACCAATCTA3'	5'GATCTCGAGTTCTGCTCTGGGAACAGTAG3'
Fragment 4	5'GATAAGCTTCACCTGTAATCCCAACTACT3'	5'GATCTCGAGAGTCCCCAAAACTGGGTGAC3'

Appendix TableS2: Sequence of 3X ERE Oligos

Forward Oligo	Reverse Complement
5'TTTGCAGGTCAGAGTGACCTGCAGGTCAGAGTGACCTGCAGGTCAGAGTGACCTGCAGGTCAGAGTGACCTGCTTT3'	5'AAAGCAGGTCACTCTGACCTGCAGGTCACTCTGACCTGCAGGTCACTCTGACCTGCAGGTCACTCTGACCTGCAAA3'

Appendix TableS3: Primers used for cloning of RRGG>AAAA ESR1

ESR1	Forward Primer	Reverse Primer
Set1	5'ATAGAATTCAACCATGACCCTCCACACCAAAGC3'	5'TTGTGTTTCAACATTCTCGCTGCTGCTGCGTCTTTTCGTATCCCACCTTTCATC3'
Set2	5'GCGCCAGAGAGATGATGGGGAGGGCAGGGGTGAAGTGGGGTCTGCTGGAGACATG3'	5'ATAGGATCCTCAGACCGTGGCAGGGAAACCCT3'

Appendix TableS4: Short hairpin RNA primers used for cloning in pLKO.1 TRC vector

shRNA	Forward Primer	Reverse Primer
shESR1 3'UTR-A	5'CCGGGCAGGATTGTTGTGGCTACTACTCGAGTAGTAGCCACAACAATCCTGCTTTTTG3'	5'AATTCAAAAAGCAGGATTGTTGTGGCTACTACTCGAGTAGTAGCCACAACAATCCTGC3'
shESR1 3'UTR-B	5'CCGGCCCTCATCATGCACCACTTTACTCGAGTAAAGTGGTGCATGATGAGGGTTTTTG3'	5'AATTCAAAAACCCTCATCATGCACCACTTTACTCGAGTAAAGTGGTGCATGATGAGGG3'
TFFeRNAshSENSE1	5'CCGGACAGGAGGTCCTCACTTA	5'AATTCAAAAAACAGGAGGT

	ATACTCGAGTATTAAGTGAGGACCTCCTGTTTTTG3'	CCTCACTTAATACTCGAGTATTAAGTGAGGACCTCCTGT3'
TFFeRNAshSENSE2	5'CCGGTCCTTGCTTGCAATGTAATTTCTCGAGAAATTACATTGCAAGCAAGGATTTTTG3'	5'AATTCAAAAATCCTTGCTTGCAATGTAATTTCTCGAGAAATTACATTGCAAGCAAGGA3'
TFFeRNAshANTISENSE1	5'CCGGGTCACTCACTCAGCGTCTTTGCTCGAGCAAAGACGCTGAGTGAGTGACTTTTTG3'	5'AATTCAAAAAGTCACTCACTCAGCGTCTTTGCTCGAGCAAAGACGCTGAGTGAGTGAC3'
TFFeRNAshANTISENSE2	5'CCGGTGTCACCTTACAGGGCAAATGCTCGAGCATTTGCCCTGTAAGGTGACATTTTTG3'	5'AATTCAAAAATGTCACCTTACAGGGCAAATGCTCGAGCATTTGCCCTGTAAGGTGACA3'
TFFeRNAshANTISENSE3	5'CCGGAGGATGTGTAAGGAGATATATCTCGAGATATATCTCCTTACACATCCTTTTTTG3'	5'AATTCAAAAAAGGATGTGTAAGGAGATATATCTCGAGATATATCTCCTTACACATCCT3'
TFFeRNAshANTISENSE4	5'CCGGCACAACAGGCCGTTCTGTATTCTCGAGAATACAGAACGGCCTGTTGTGTTTTTG3'	5'AATTCAAAAACACAACAGGCCGTTCTGTATTCTCGAGAATACAGAACGGCCTGTTGTG3'
shScramble	5'CCGGACAACCGCGAAAAAGTTGCGCCTCGAGGCGCAACTTTTTCGCGGTTGTTTTTTG3'	5'AATTCAAAAAACAACCGCGAAAAAGTTGCGCCTCGAGGCGCAACTTTTTCGCGGTTGT3'

Appendix TableS5: Primers used for ChIPqPCR and RTqPCR

Name	Forward Primer	Reverse Primer
TFF1enhancer ChIP	5’GCAGCCAGGAAAAGGAGTGA3’	5’ACGTGTACGGTGGCATCATC3’
FOXC1enhancer ChIP	5'CTGAGGAACACAAGACTAGCC3'	5'ACTGGACTCATTTTGGGACATC3'
GREB1enhancer ChIP	5’AATGGGAGTGATCTGAGTGGTT3’	5’TTTCATGTATGAGGCAATGGTC3’
NRIP1enhancer ChIP	5’GGCTCGTCATTAGCTTCCAG3’	5’CAAAAAGGGGTGTTGAAGGA3’
GREB1promoter ChIP	5’CAGCTGACTGTCTTCCACCA3’	5’CCACCGTTTCGTGTCTTCTT3’
Luciferase qRT	5’CGTGAGCAAGAAAGGGCTGC3’	5’CGATGGTTTTGTCCCGGTCG3’

Appendix TableS6: Accession number of NGS Dataset

Accession Number	Experiment	Reference
GSM1702269	chrRNA-seq	Ntini E et al., 2018
GSM2978808	NPRNA-seq	Ntini E et al., 2018
GSM1115990	ERα ChIP-seq Repeat 1	Li W, Notani D. et al., 2013
GSM1115991	ERα ChIP-seq Repeat 2	Li W, Notani D. et al., 2013
GSM4006678	MCF-7 WT ERα ChIP-seq	Yang M et al., 2020
GSM4006672	MCF-7 Δ ERα ChIP-seq	Yang M et al., 2020
GSM4006681	MCF-7 BLRP WT ERα	Yang M et al., 2020

GSM4006680	MCF-7 BLRP pbox ERα	Yang M et al., 2020
GSM4259508	MCF-7 EtOH TT-seq Repeat 1	Lee JH et al., 2021
GSM4259512	MCF-7 EtOH TT-seq Repeat 2	Lee JH et al., 2021
GSM4259510	MCF-7 E2 TT-seq Repeat 1	Lee JH et al., 2021
GSM4259514	MCF-7 E2 TT-seq Repeat 2	Lee JH et al., 2021
GSM3863288	MCF-7 H3k27ac	Jiang G et al., 2019
GSM5281991	T47D DMSO ERα ChIP-seq repeat 1	Zhang Y et al., 2021
GSM5282005	T47D DMSO ERα ChIP-seq repeat 2	Zhang Y et al., 2021
GSM5281995	T47D Triptolide ERα ChIP-seq repeat 1	Zhang Y et al., 2021
GSM5282008	T47D Triptolide ERα ChIP-seq repeat 2	Zhang Y et al., 2021
GSM5176099	MCF-7 total Pol-II veh repeat 1	Sun J et al., 2021
GSM5176100	MCF-7 total Pol-II veh repeat 2	Sun J et al., 2021
GSM5176101	MCF-7 total Pol-II E2 repeat1	Sun J et al., 2021
GSM5176102	MCF-7 total Pol-II E2 repeat1	Sun J et al., 2021
GSM7719569	MCF-7 Veh ERα fRIP-seq	This study
GSM7719570	MCF-7 Veh Input fRIP-seq	
GSM7719565	MCF-7 E2 ERα fRIP-seq Repeat 1	This study
GSM7719566	MCF-7 E2 Input fRIP-seq Repeat 1	
GSM7719567	MCF-7 E2 ERα fRIP-seq Repeat 2	This study
GSM7719568	MCF-7 E2 Input fRIP-seq Repeat 2	
GSM7719571	MCF-7 E2 WT FLAG ChIP-seq Repeat 1	This study
GSM7719572	MCF-7 E2 WT FLAG ChIP-seq Repeat 2	This study
GSM7719573	MCF-7 E2 RBM FLAG ChIP-seq Repeat 1	This study
GSM7719574	MCF-7 E2 RBM FLAG ChIP-seq Repeat 2	This study
GSM7719583	MCF-7 E2 WT FLAG and CTCF ChIP-seq	This study
GSM7719584	MCF-7 E2 RBM FLAG and CTCF ChIP-seq	This study
GSM7719579	MCF-7 E2 Mock w/o pre-extraction ERα ChIP-seq Repeat 1	This study
GSM7719580	MCF-7 E2 Mock w/o pre-extraction ERα ChIP-seq Repeat 2	This study

GSM7719581	MCF-7 E2 RNAse A w/o pre-extraction ERα ChIP-seq Repeat 1	This study
GSM7719582	MCF-7 E2 RNAse A w/o pre-extraction ERα ChIP-seq Repeat 2	This study
GSM7719575	MCF-7 E2 Mock w pre-extraction ERα ChIP-seq Repeat 1	This study
GSM7719576	MCF-7 E2 Mock w pre-extraction ERα ChIP-seq Repeat 2	This study
GSM7719577	MCF-7 E2 RNAse A w pre-extraction ERα ChIP-seq Repeat 1	This study
GSM7719578	MCF-7 E2 RNAse A w pre-extraction ERα ChIP-seq Repeat 2	This study
GSM7719587	MCF-7 E2 WT EU-seq Repeat 1	This study
GSM7719589	MCF-7 E2 WT EU-seq Repeat 2	This study
GSM7719588	MCF-7 E2 RBM EU-seq Repeat 1	This study
GSM7719590	MCF-7 E2 RBM EU-seq Repeat 2	This study
GSM7719585	MCF-7 E2 WT Total PolII	This study
GSM7719586	MCF-7 E2 RBM Total PolII	This study
