# Supplementary figures and images for: RNA fine-tunes estrogen receptor-alpha binding on low-affinity DNA motifs for transcriptional regulation
Source: EMBO J. 2024 Sep 16;43(21):5186–210. doi: 10.1038/s44318-024-00225-y (PMC11535219; doi:10.1038/s44318-024-00225-y)

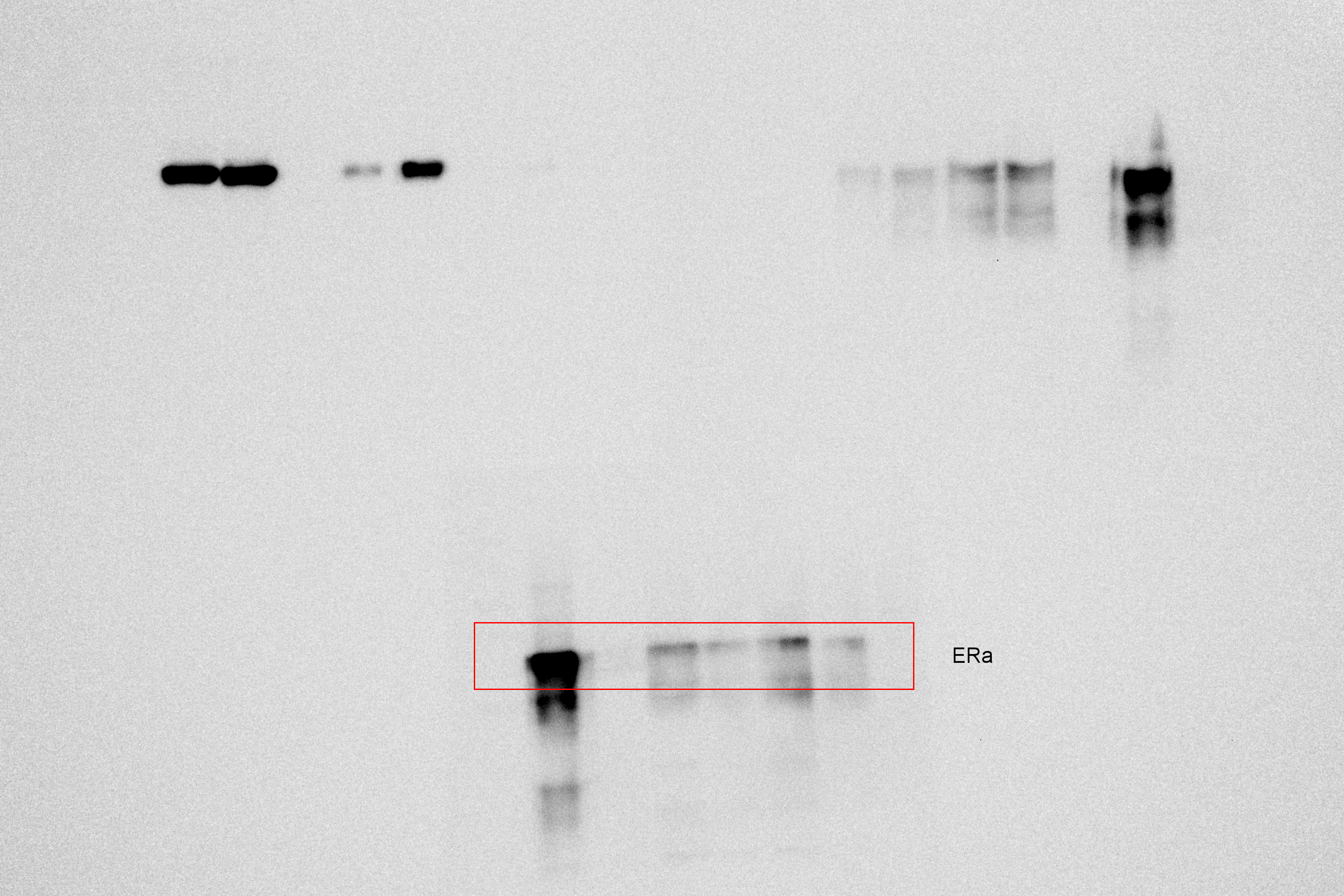

Supplement: Supplementary file 3 — Source data Fig. 1 [file 44318_2024_225_MOESM3_ESM.zip › Figure_1_sourcedatafile/1F/ERaWB_1F.tif]

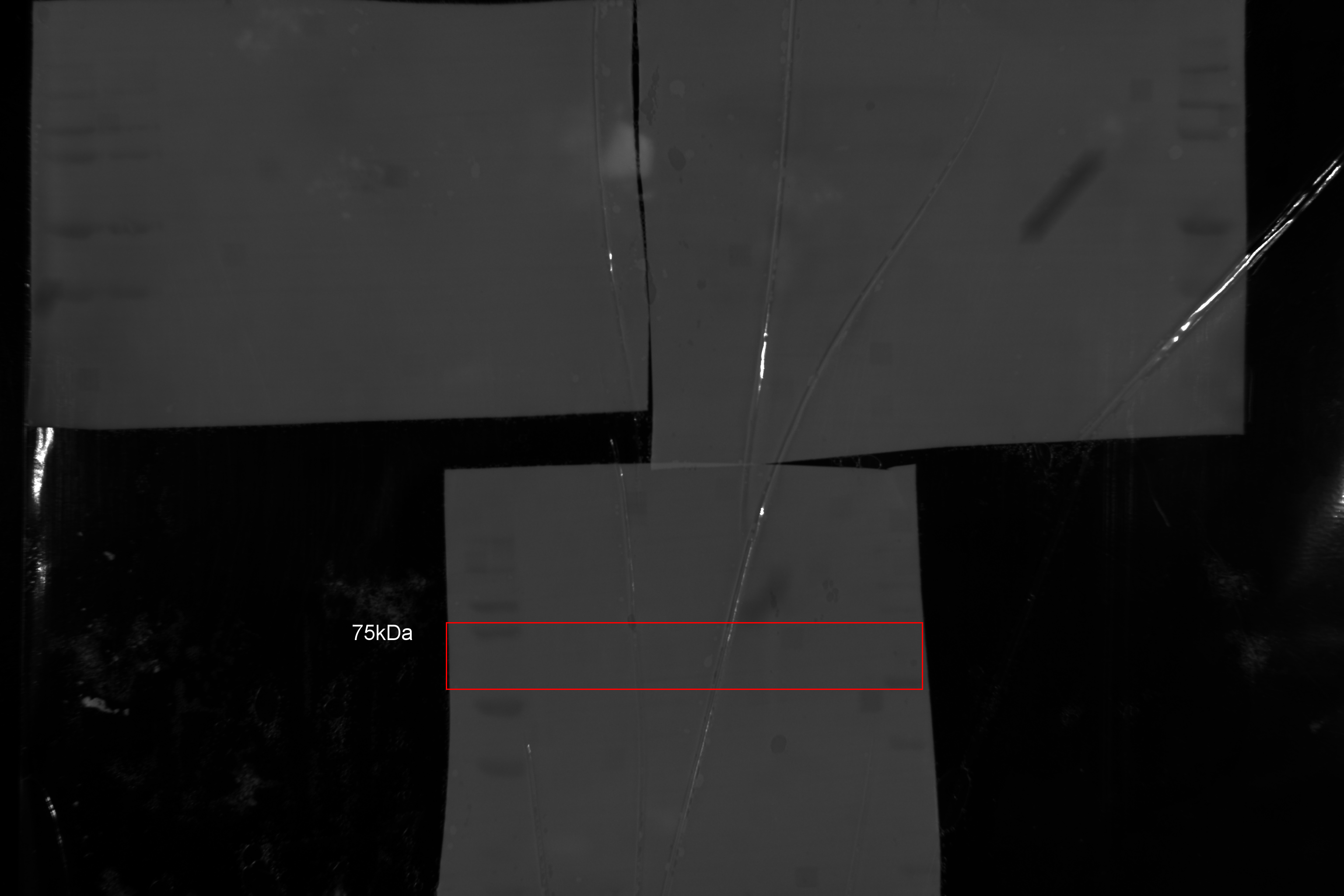

Supplement: Supplementary file 3 — Source data Fig. 1 [file 44318_2024_225_MOESM3_ESM.zip › Figure_1_sourcedatafile/1F/Molecularmarker_1F.tif]

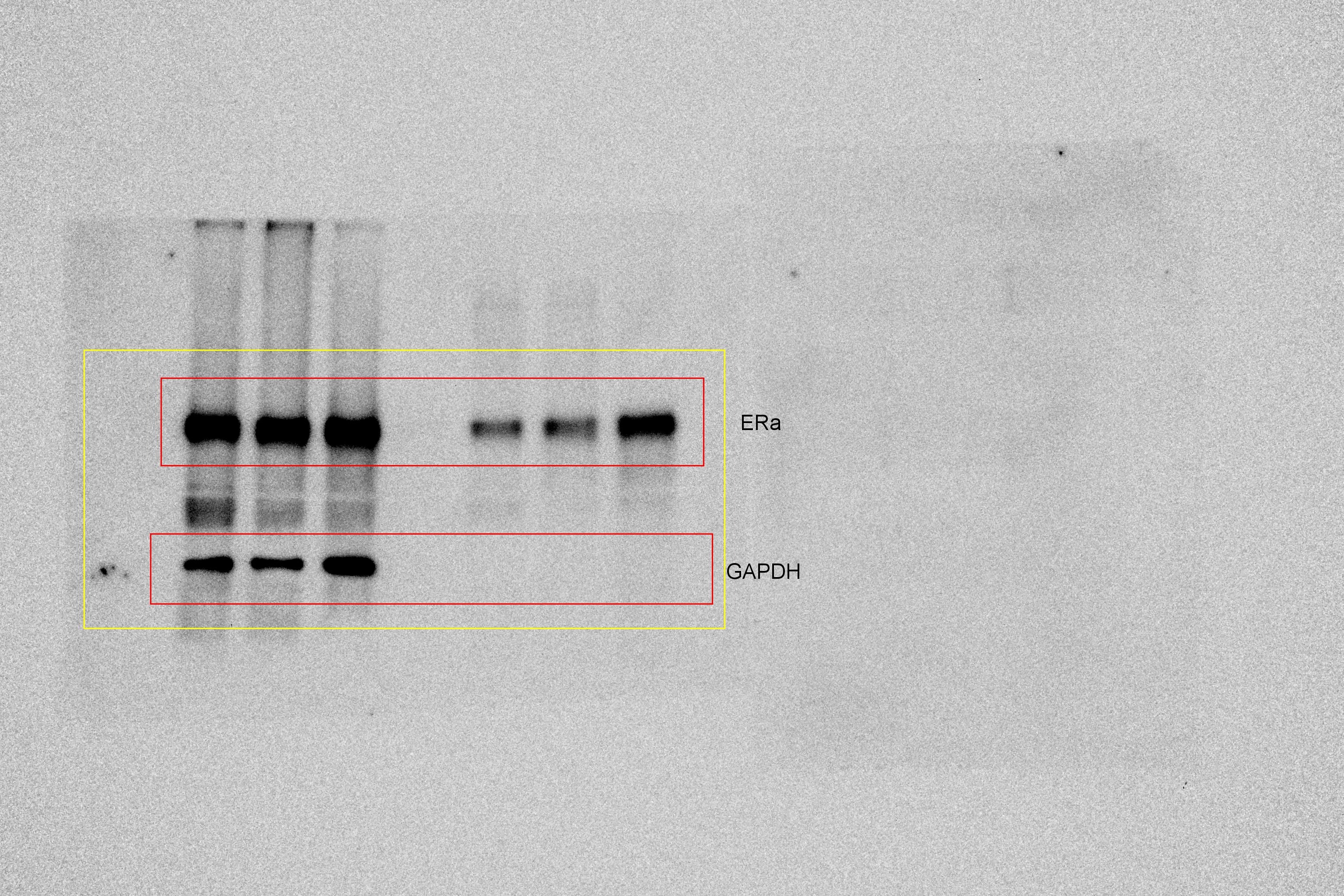

Supplement: Supplementary file 3 — Source data Fig. 1 [file 44318_2024_225_MOESM3_ESM.zip › Figure_1_sourcedatafile/1G/ERaandGAPDHWB_1G.tif]

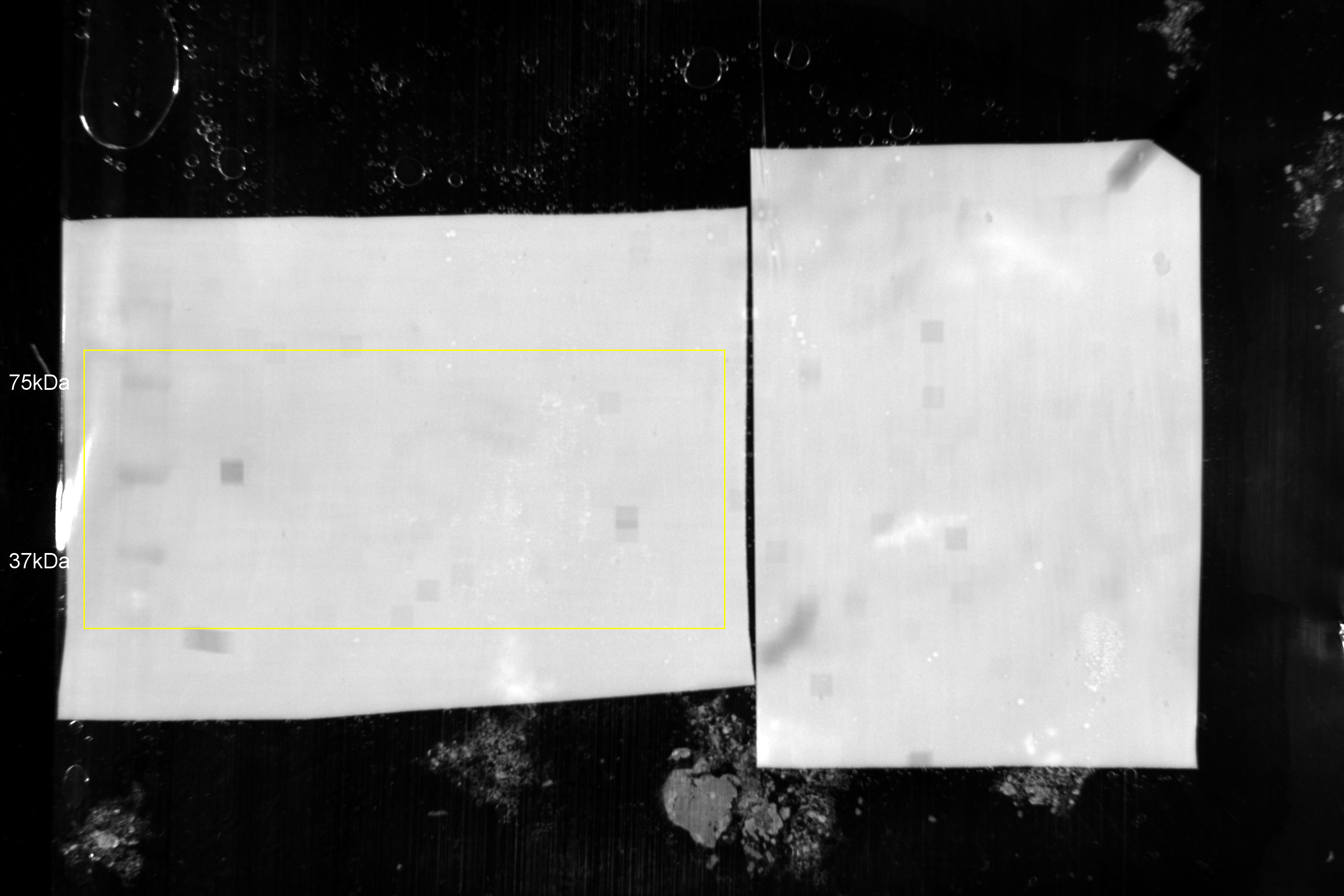

Supplement: Supplementary file 3 — Source data Fig. 1 [file 44318_2024_225_MOESM3_ESM.zip › Figure_1_sourcedatafile/1G/Molecularmarker_1G.tif]

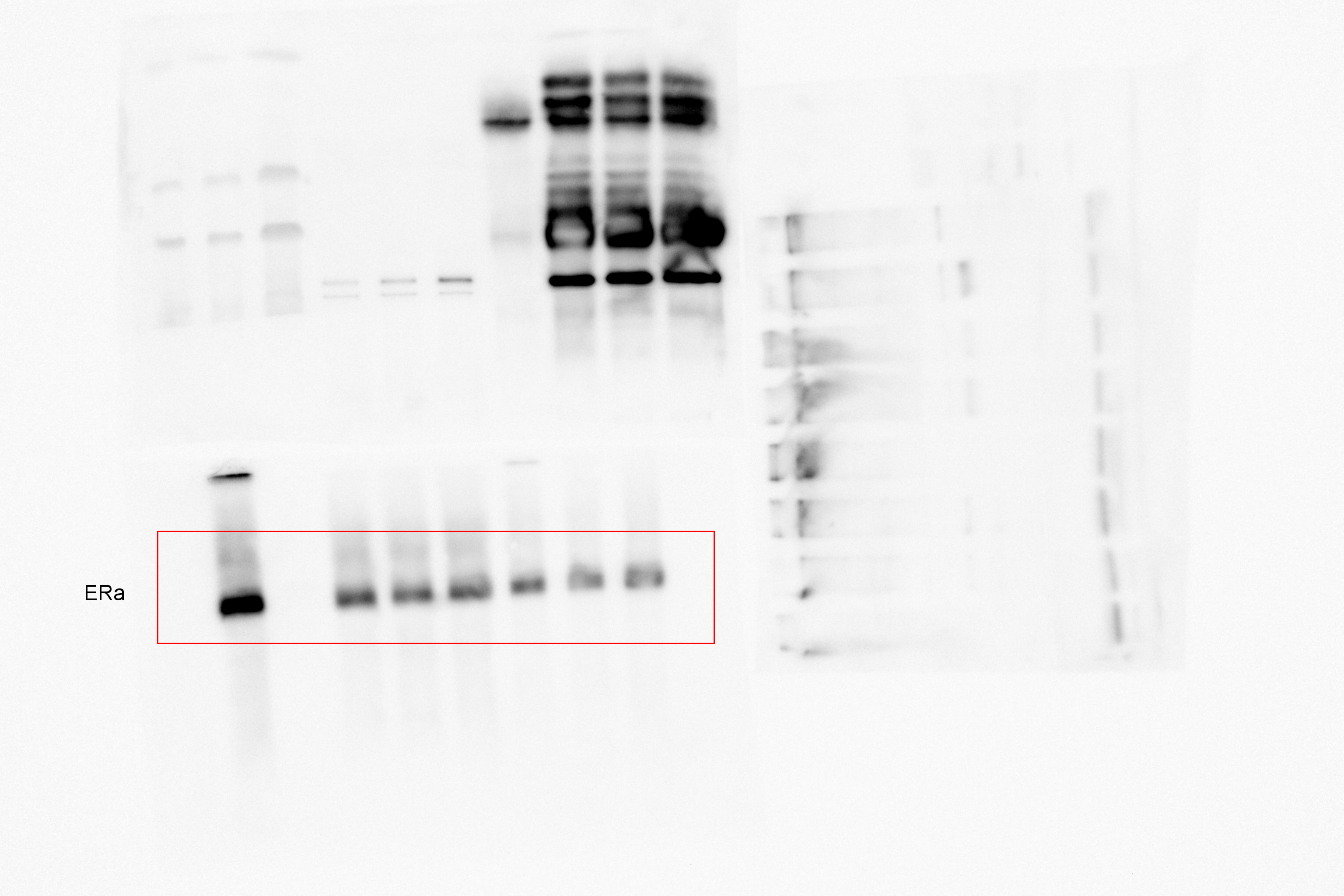

Supplement: Supplementary file 3 — Source data Fig. 1 [file 44318_2024_225_MOESM3_ESM.zip › Figure_1_sourcedatafile/1H/ERaWB_1H.tif]

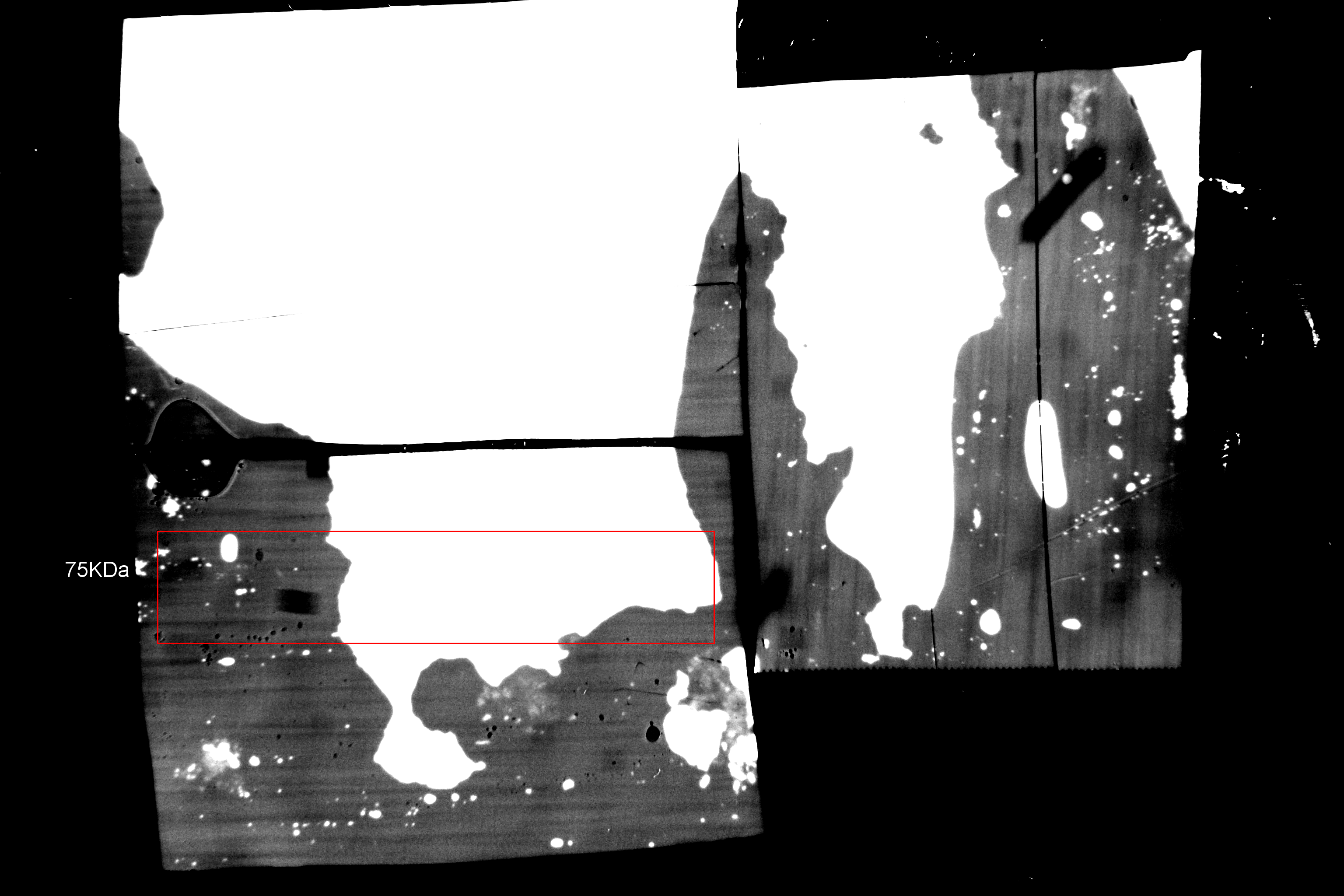

Supplement: Supplementary file 3 — Source data Fig. 1 [file 44318_2024_225_MOESM3_ESM.zip › Figure_1_sourcedatafile/1H/Molecularmarker_1H.tif]

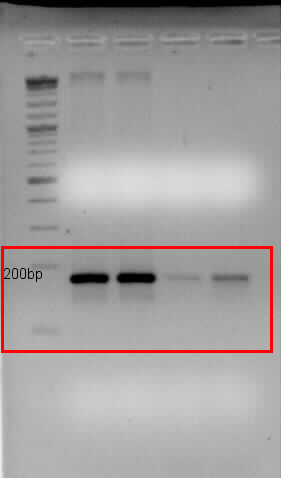

Supplement: Supplementary file 3 — Source data Fig. 1 [file 44318_2024_225_MOESM3_ESM.zip › Figure_1_sourcedatafile/1I/Agarosegelimage_DNAladder_PCRproduct_1I.tif]

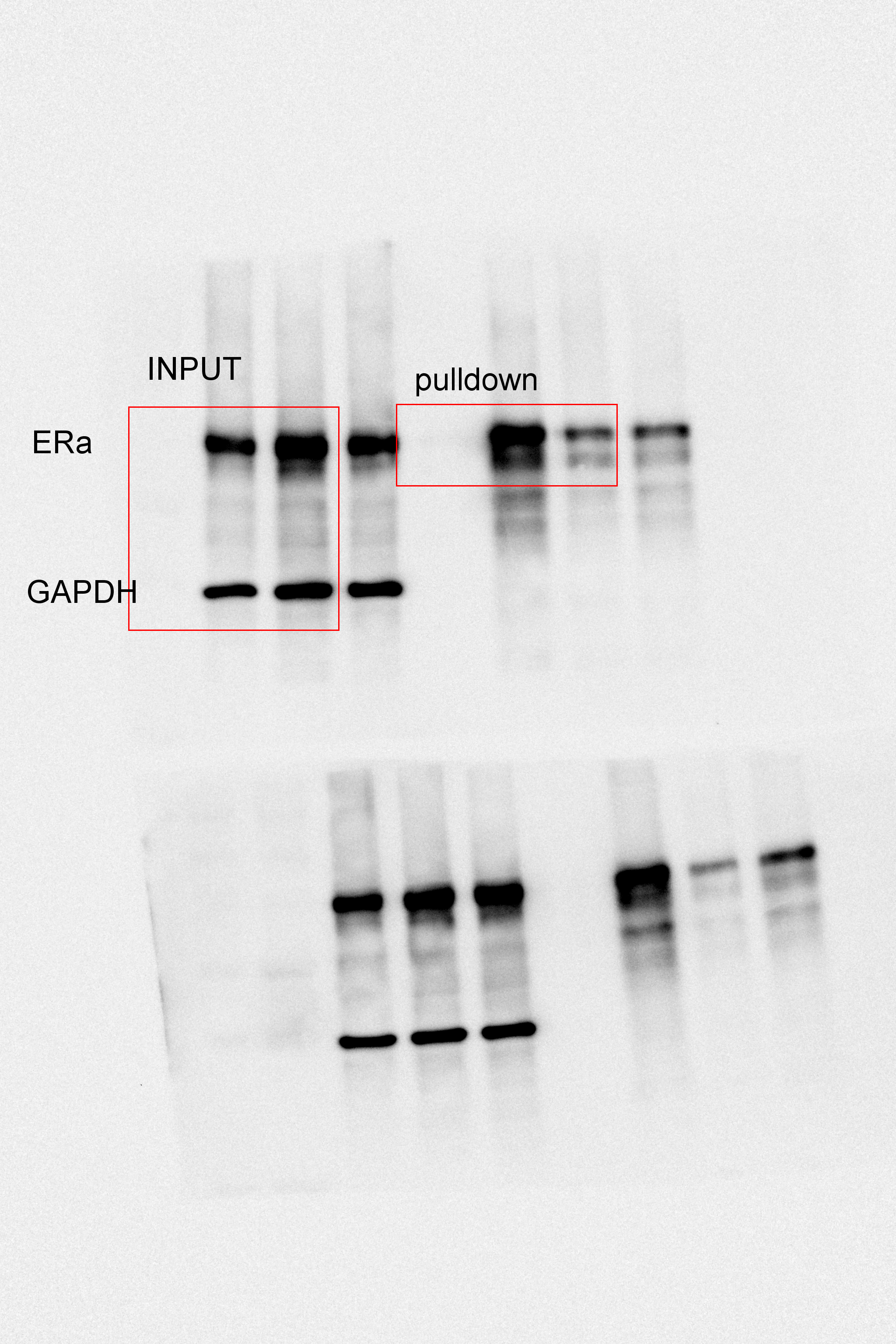

Supplement: Supplementary file 4 — Source data Fig. 2 [file 44318_2024_225_MOESM4_ESM.zip › Figure_2_sourcedatafile/2A/ERaandGAPDH_2A.tif]

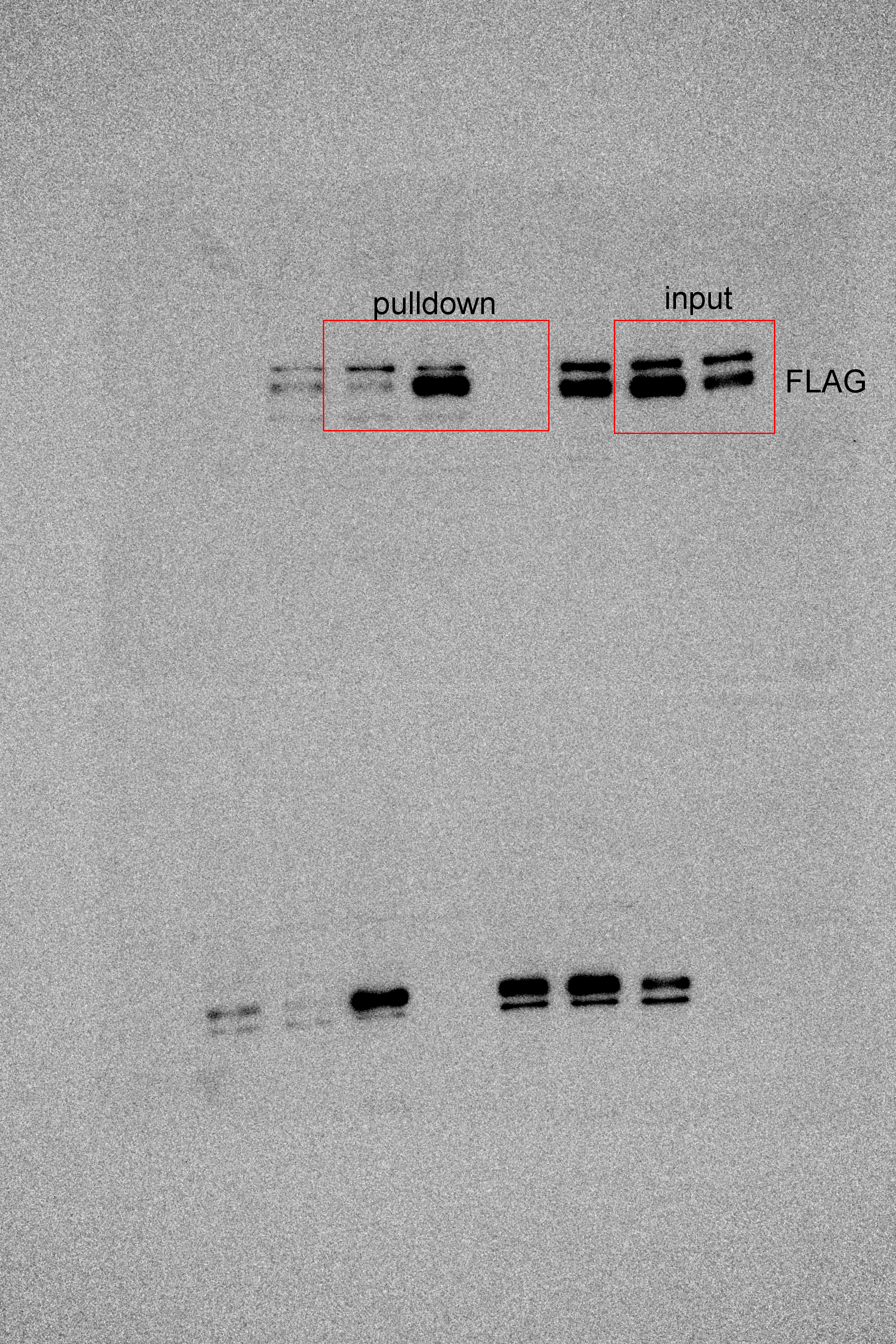

Supplement: Supplementary file 4 — Source data Fig. 2 [file 44318_2024_225_MOESM4_ESM.zip › Figure_2_sourcedatafile/2A/FLAGWB_2A.tif]

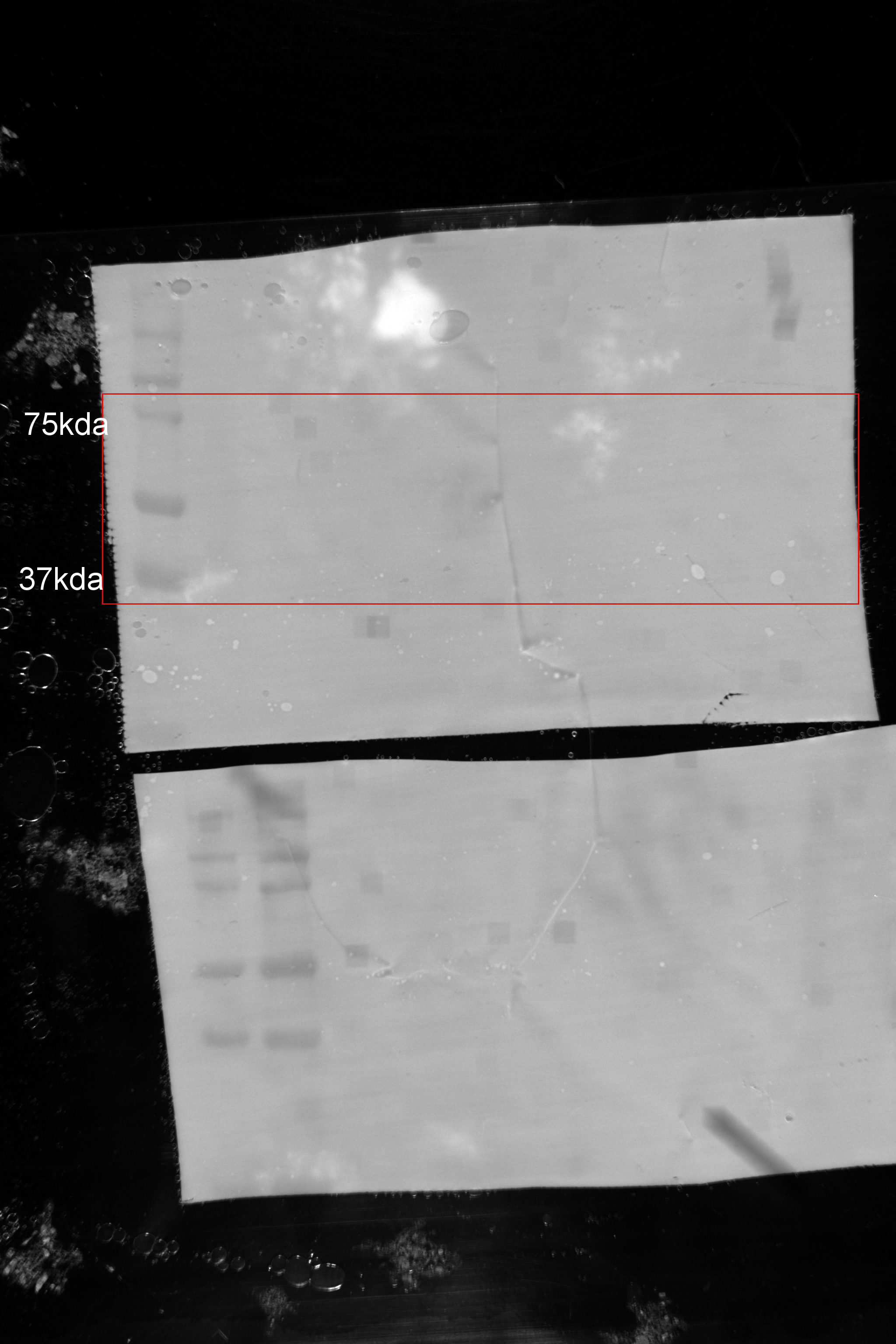

Supplement: Supplementary file 4 — Source data Fig. 2 [file 44318_2024_225_MOESM4_ESM.zip › Figure_2_sourcedatafile/2A/MolecularmarkerERaGAPDH_2A.tif]

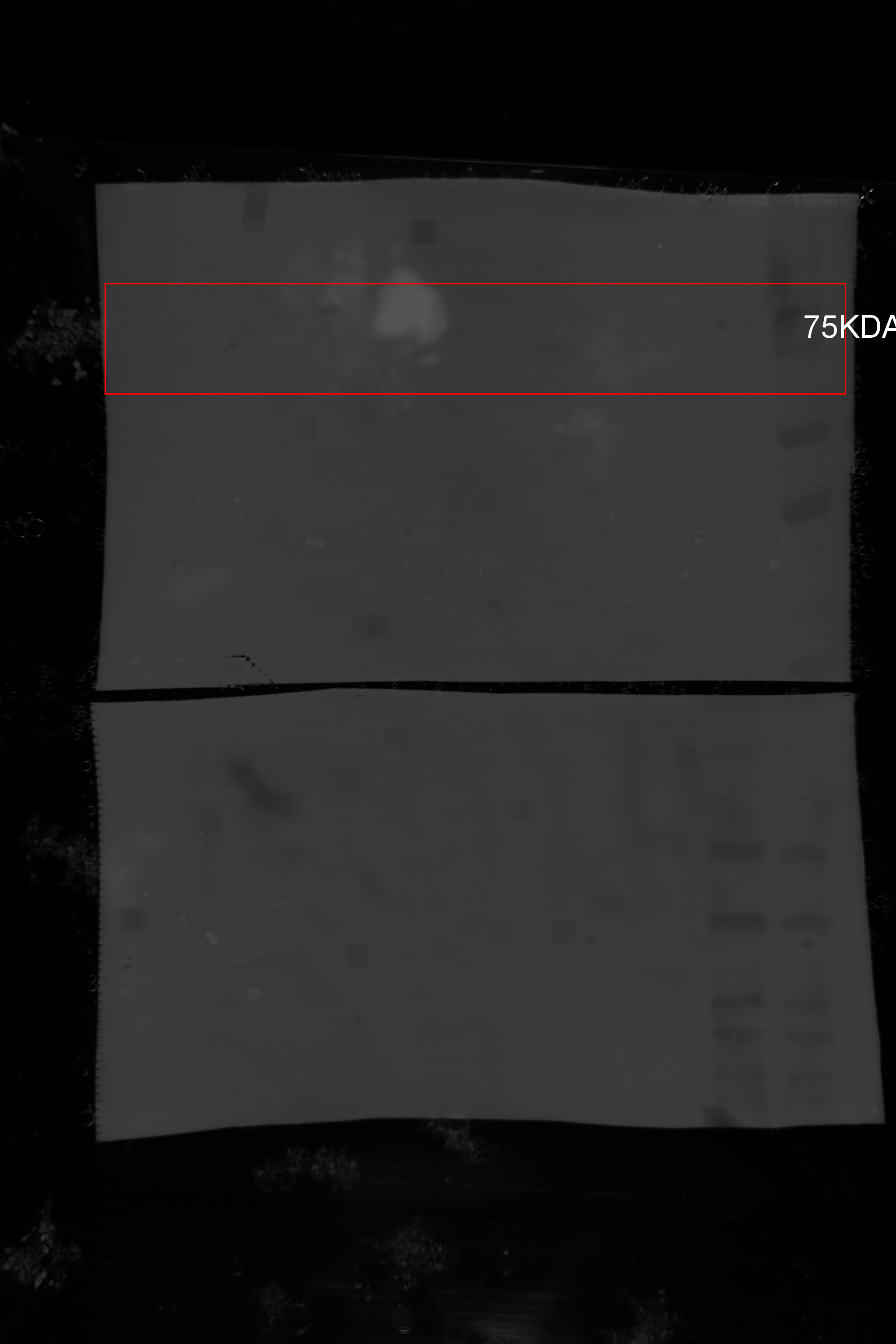

Supplement: Supplementary file 4 — Source data Fig. 2 [file 44318_2024_225_MOESM4_ESM.zip › Figure_2_sourcedatafile/2A/MolecularmarkerFLAG_2A.tif]

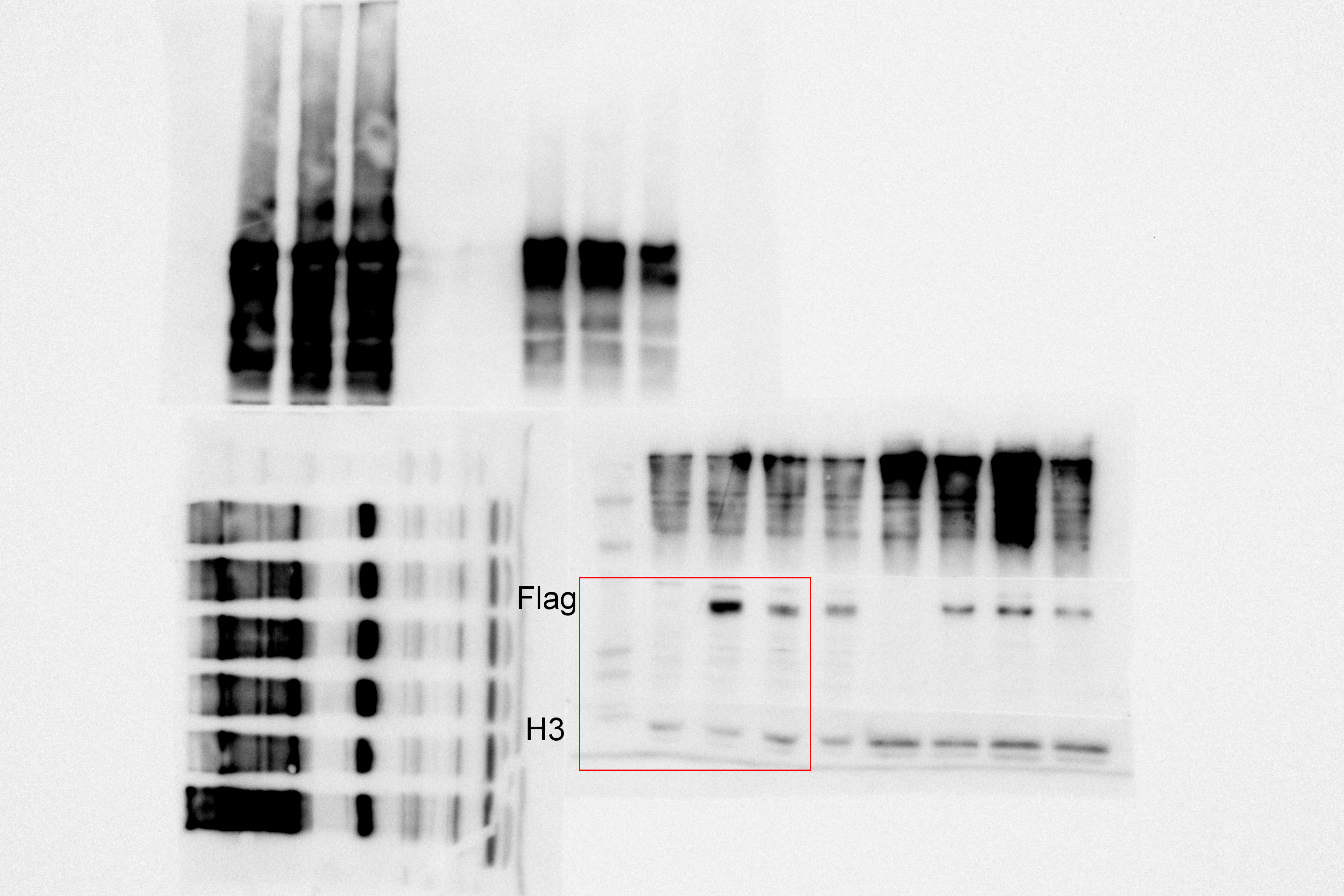

Supplement: Supplementary file 4 — Source data Fig. 2 [file 44318_2024_225_MOESM4_ESM.zip › Figure_2_sourcedatafile/2B/FLAGandH3_2B.tif]

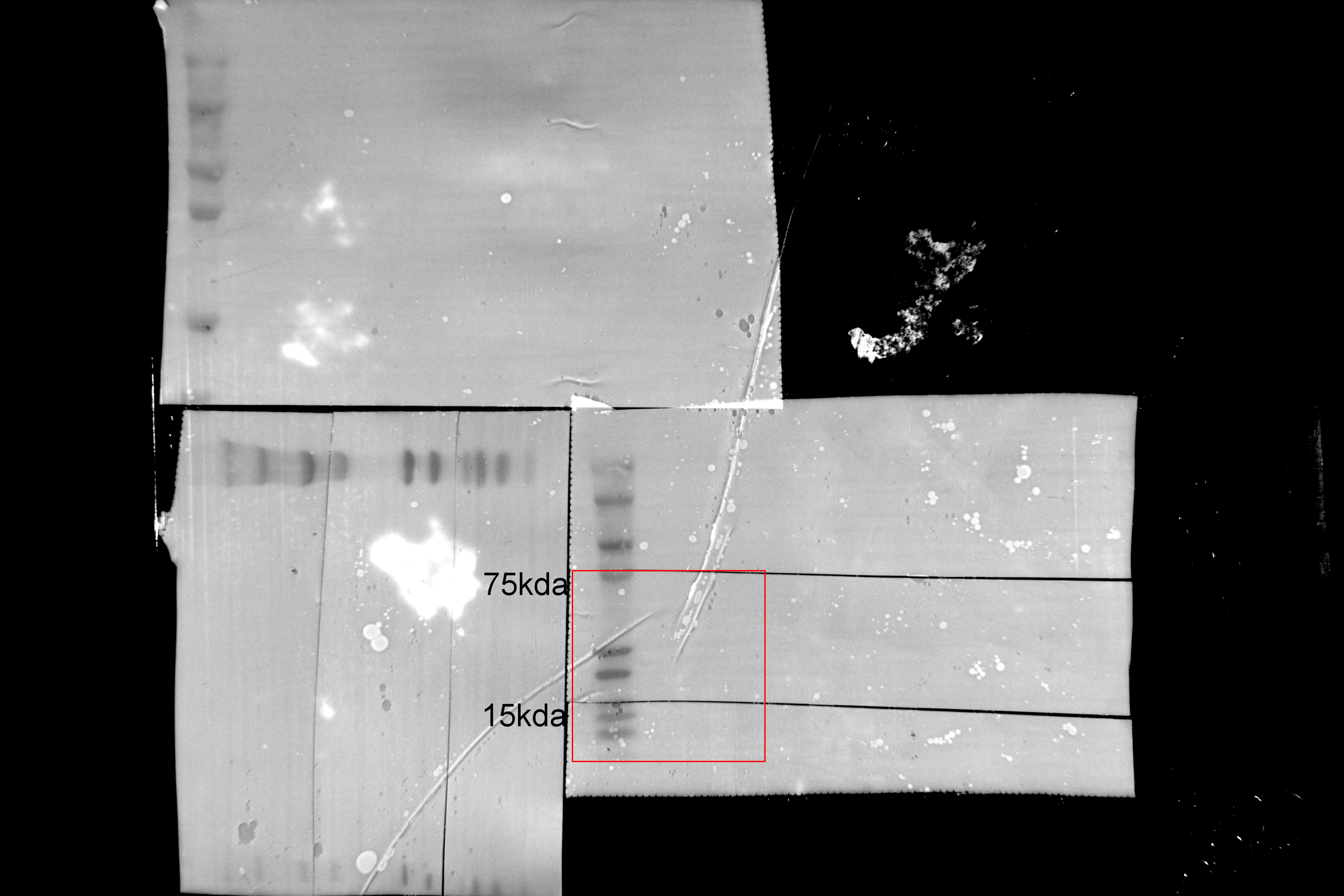

Supplement: Supplementary file 4 — Source data Fig. 2 [file 44318_2024_225_MOESM4_ESM.zip › Figure_2_sourcedatafile/2B/Molecularmarker_2B.tif]

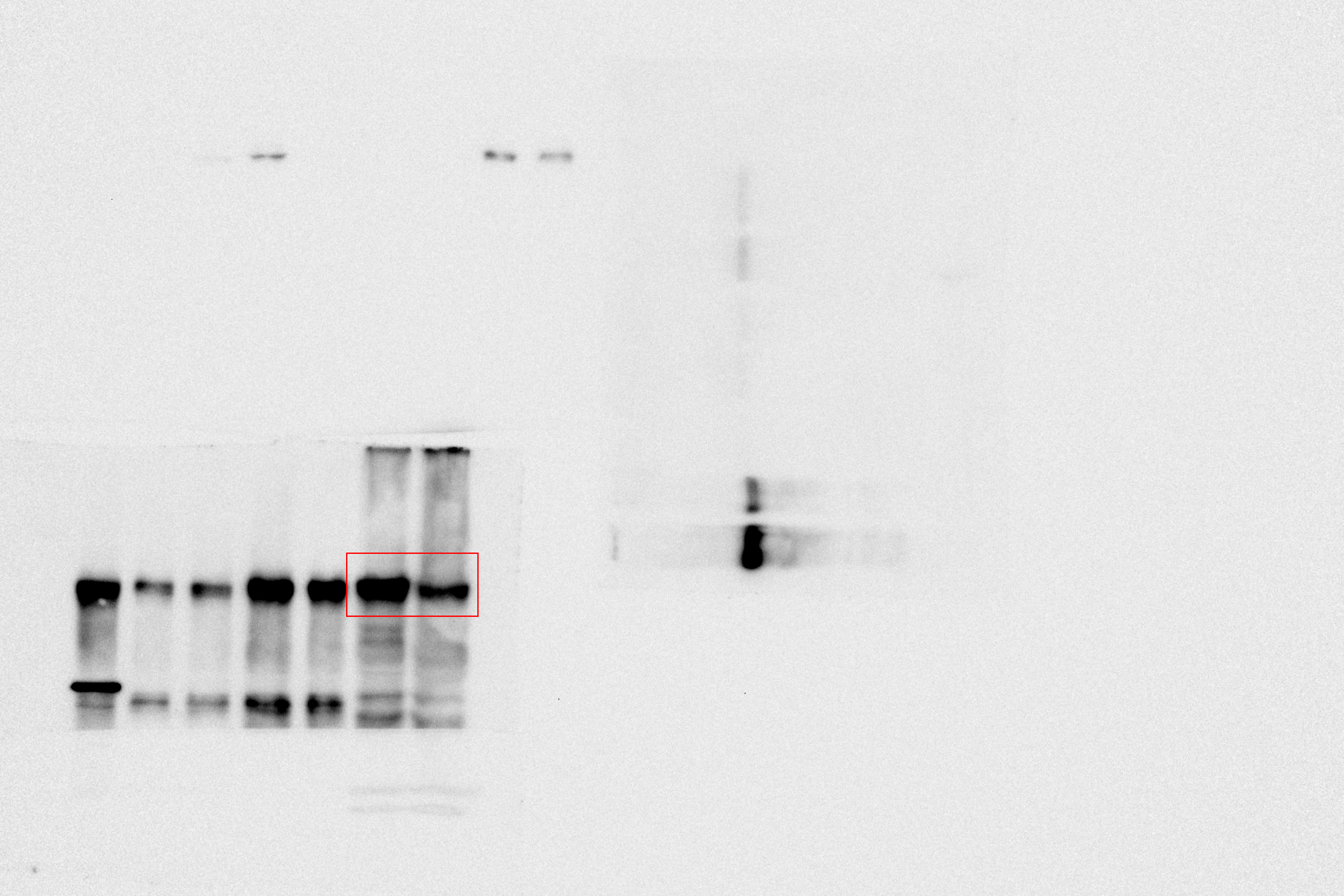

Supplement: Supplementary file 6 — Source data Fig. 4 [file 44318_2024_225_MOESM6_ESM.zip › Figure_4_sourcedatafile/4C/ERaWB.tif]

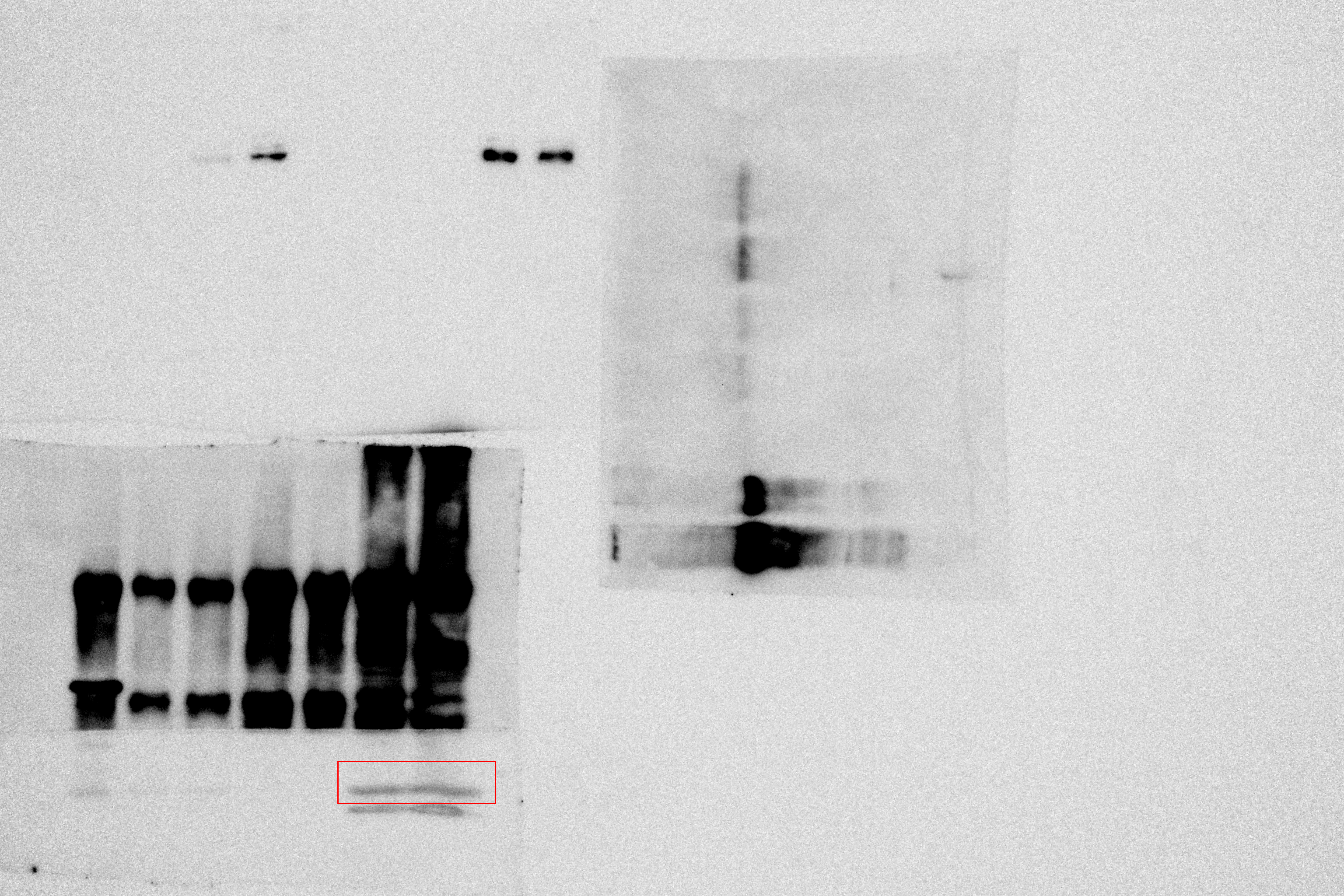

Supplement: Supplementary file 6 — Source data Fig. 4 [file 44318_2024_225_MOESM6_ESM.zip › Figure_4_sourcedatafile/4C/H3WB.tif]

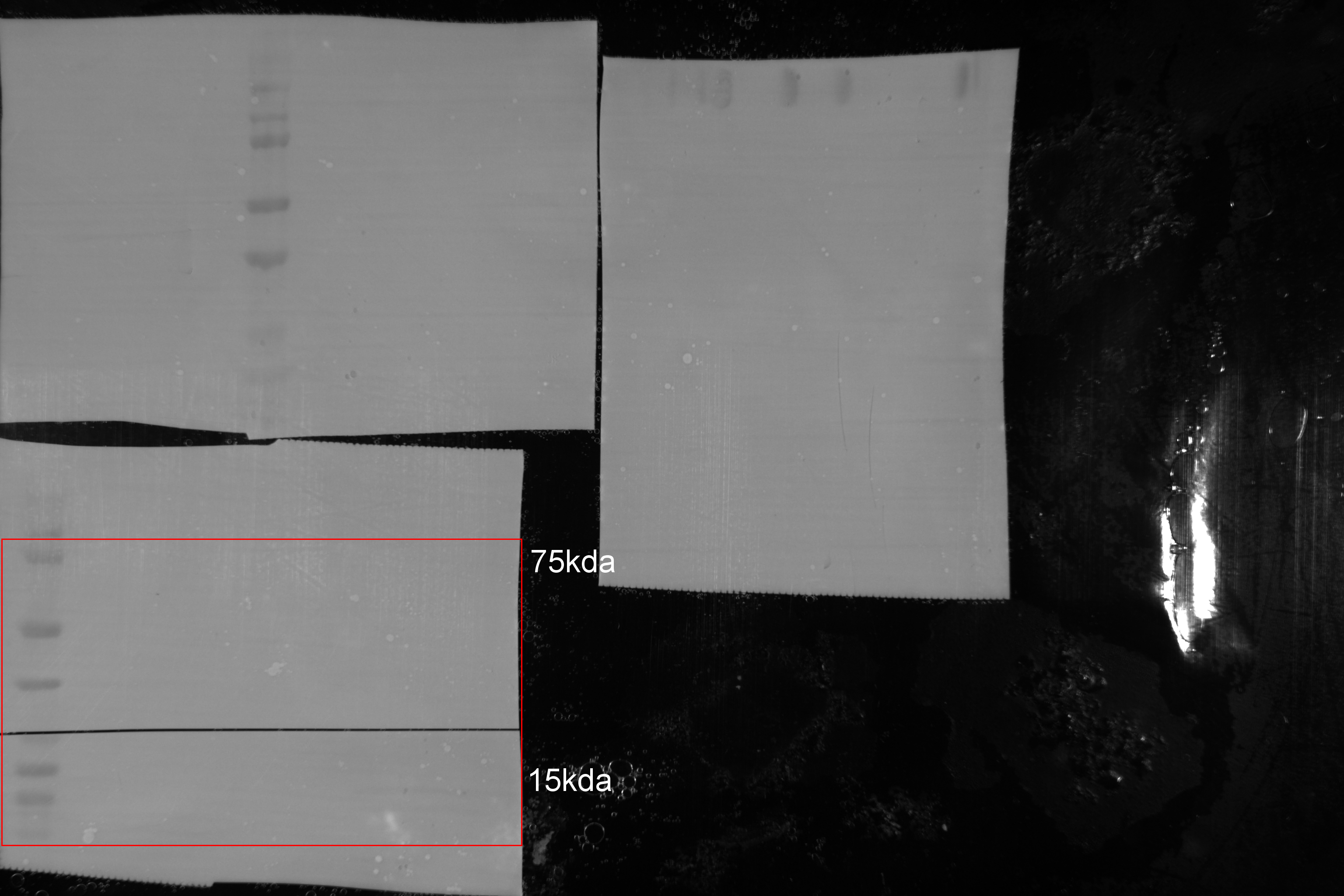

Supplement: Supplementary file 6 — Source data Fig. 4 [file 44318_2024_225_MOESM6_ESM.zip › Figure_4_sourcedatafile/4C/Molecularmarker.tif]

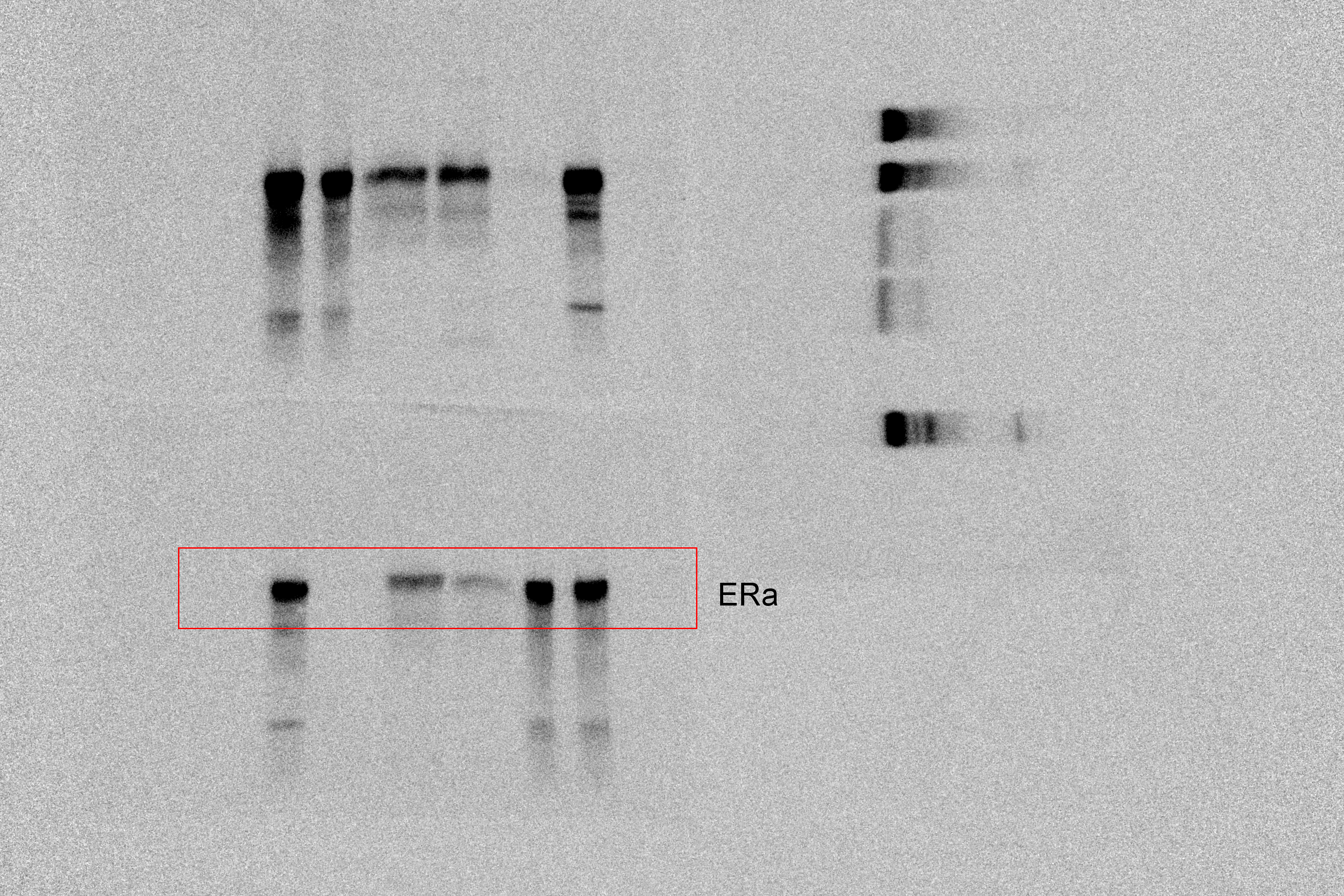

Supplement: Supplementary file 6 — Source data Fig. 4 [file 44318_2024_225_MOESM6_ESM.zip › Figure_4_sourcedatafile/4D/ERaWB.tif]

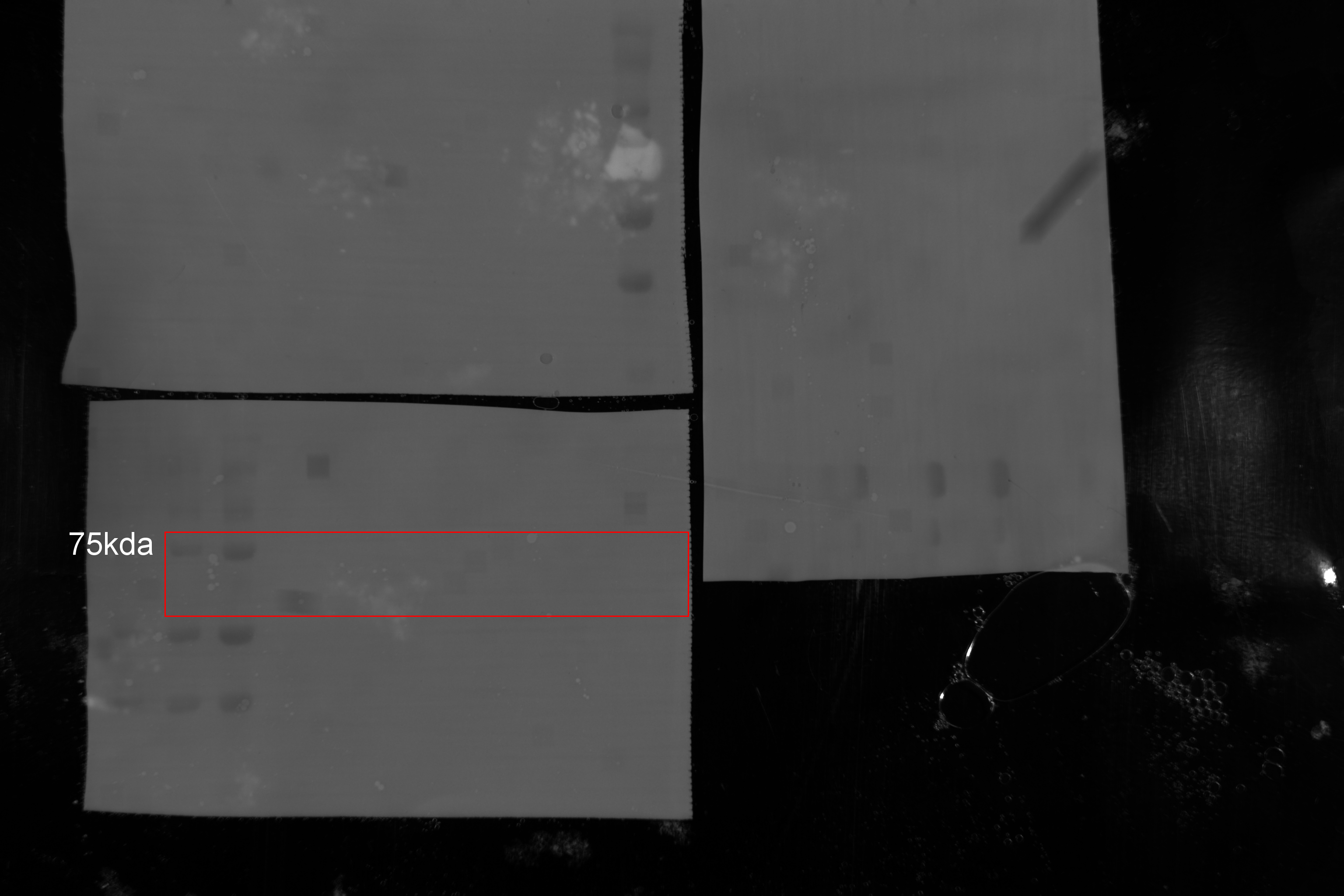

Supplement: Supplementary file 6 — Source data Fig. 4 [file 44318_2024_225_MOESM6_ESM.zip › Figure_4_sourcedatafile/4D/Molecularmarker.tif]

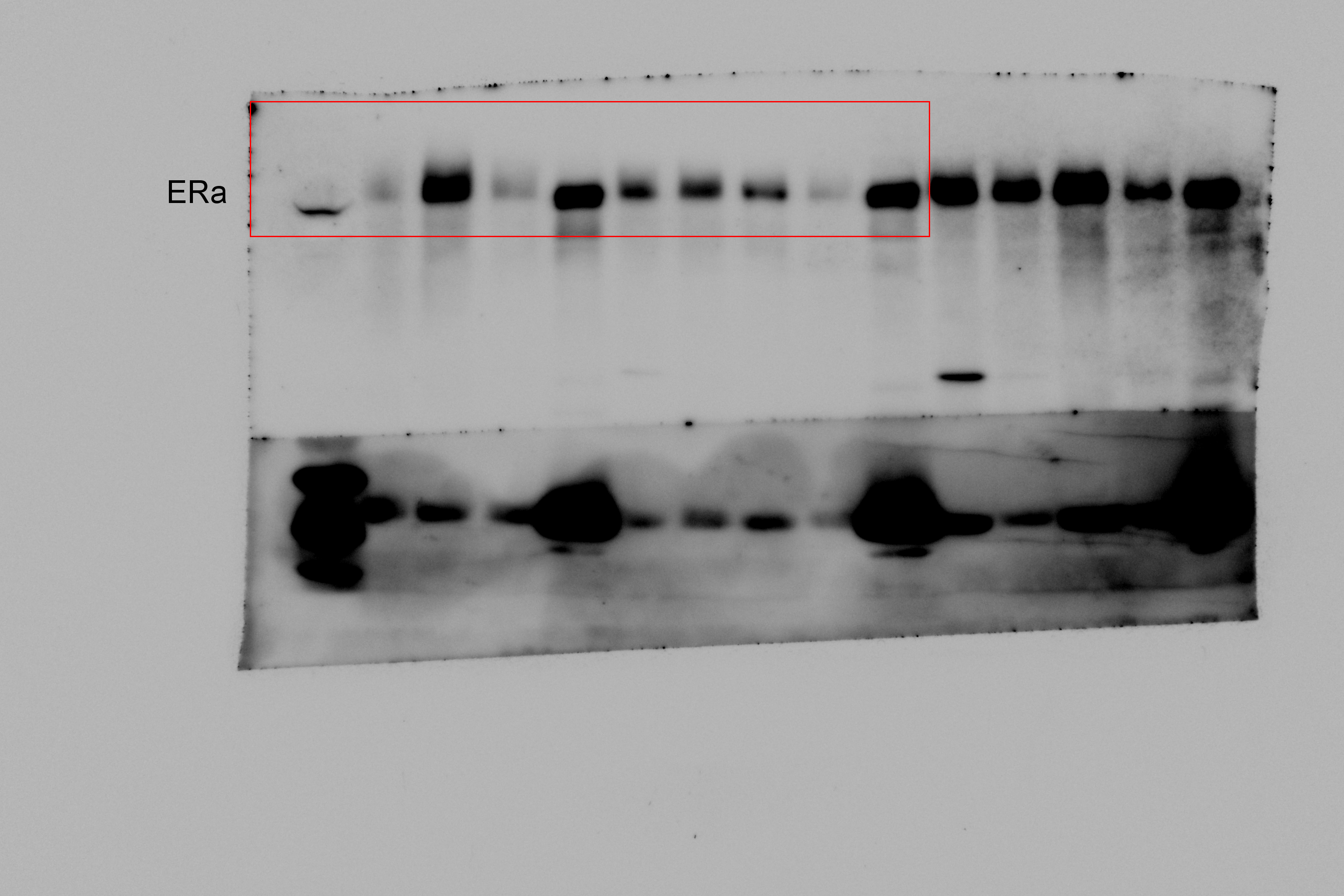

Supplement: Supplementary file 9 — Source data Fig. 5 [file 44318_2024_225_MOESM9_ESM.zip › Figure_5_sourcedatafile/5F/ERaWB.tif]

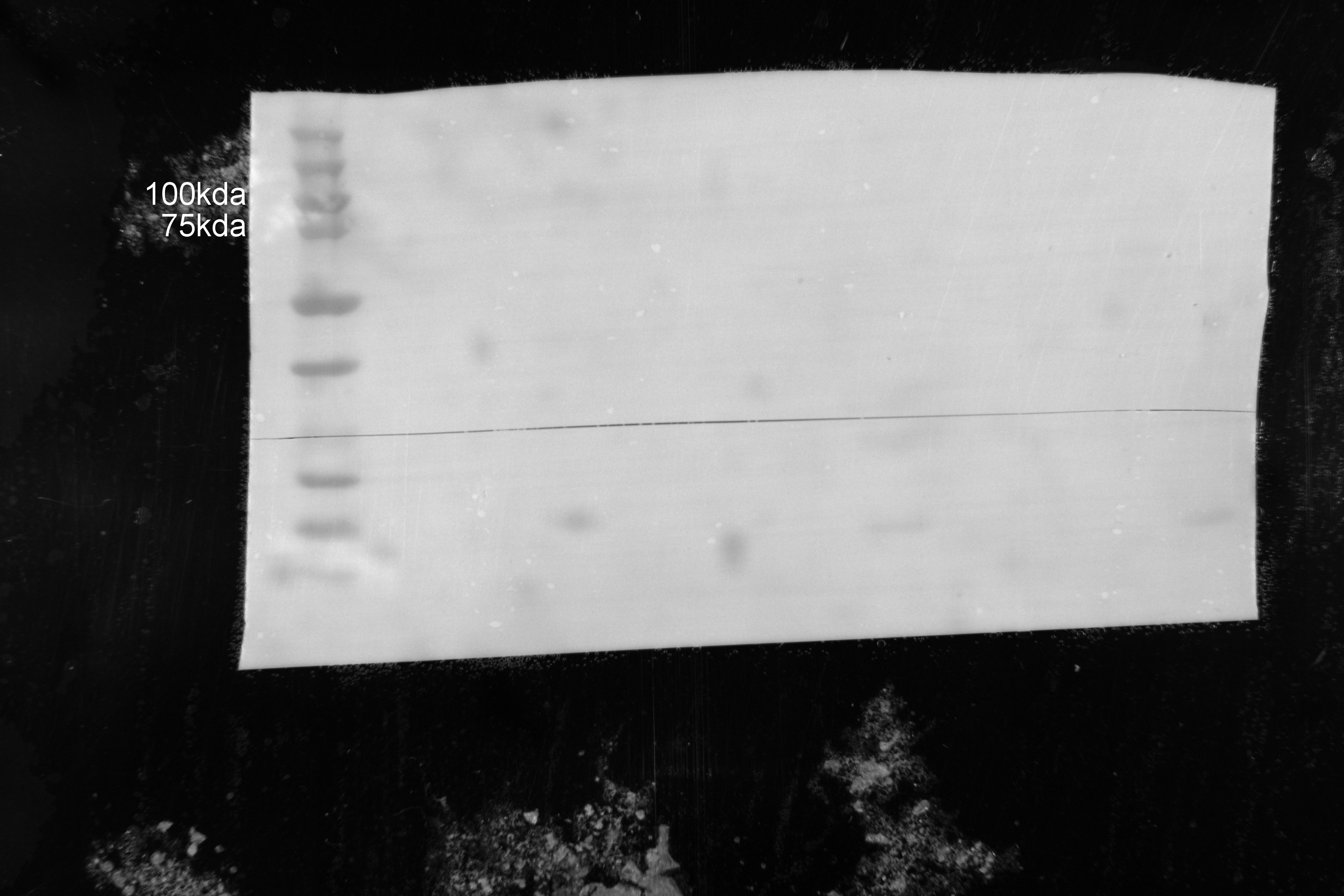

Supplement: Supplementary file 9 — Source data Fig. 5 [file 44318_2024_225_MOESM9_ESM.zip › Figure_5_sourcedatafile/5F/Molecularmarker.tif]

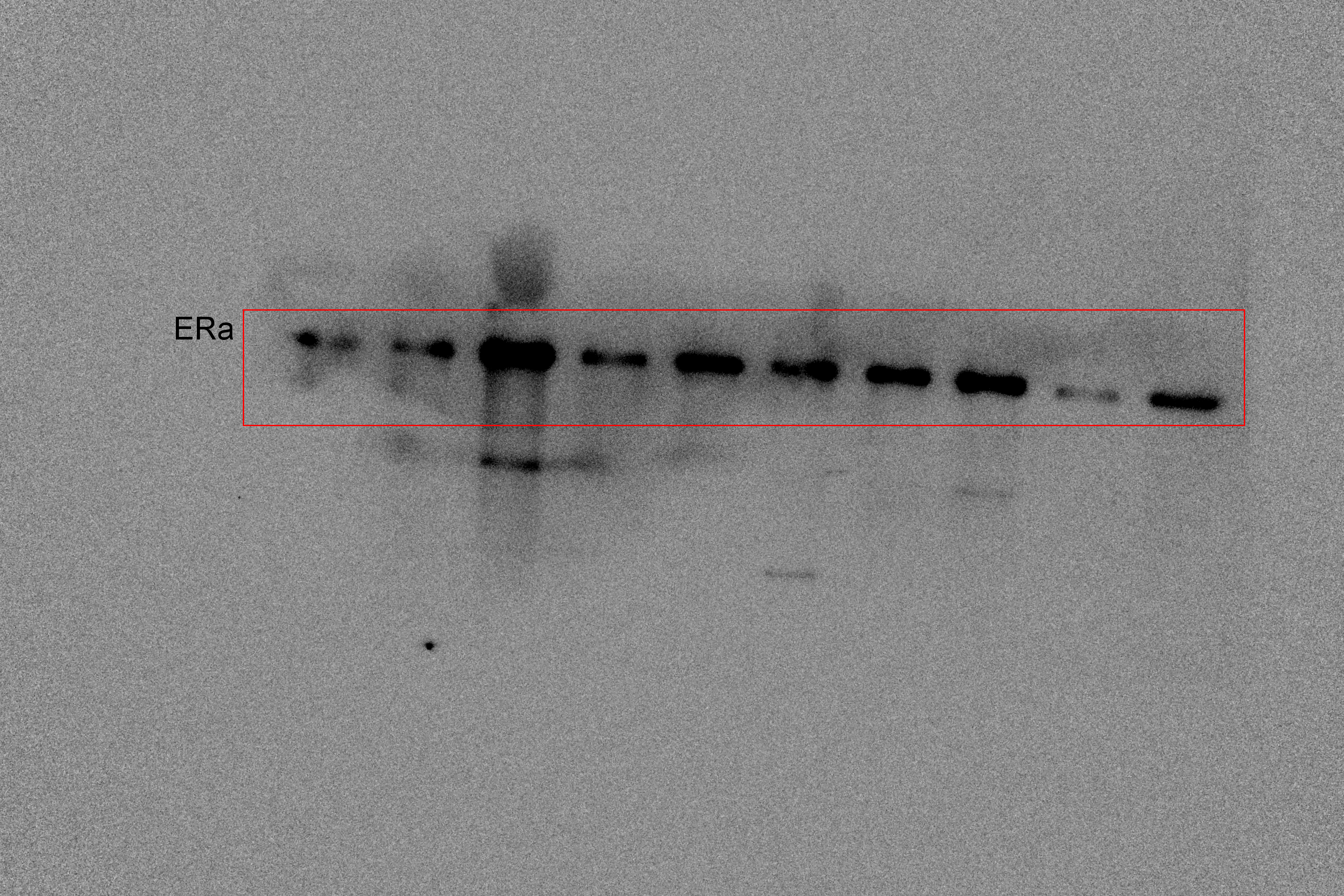

Supplement: Supplementary file 9 — Source data Fig. 5 [file 44318_2024_225_MOESM9_ESM.zip › Figure_5_sourcedatafile/5G/ERaWB.tif]

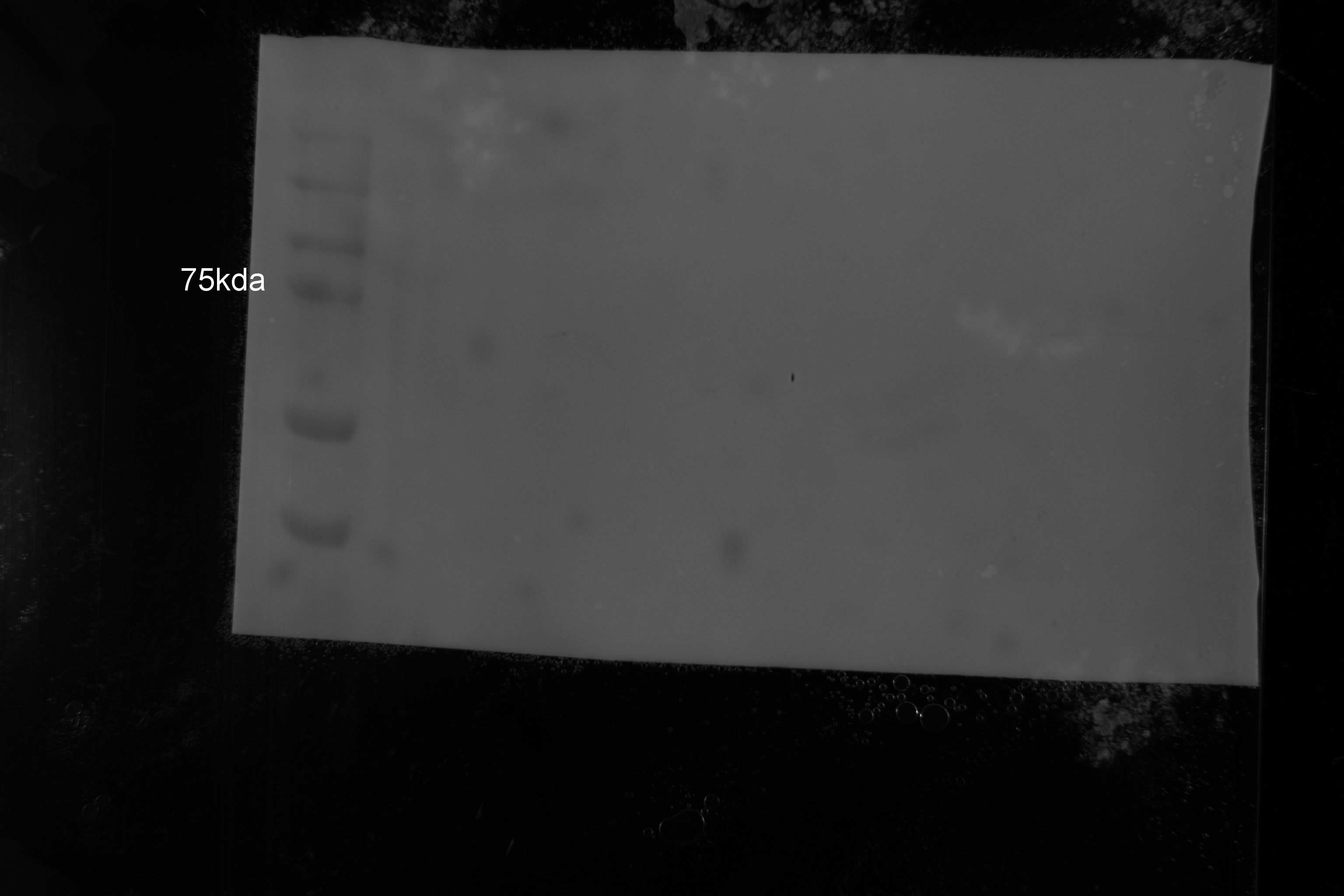

Supplement: Supplementary file 9 — Source data Fig. 5 [file 44318_2024_225_MOESM9_ESM.zip › Figure_5_sourcedatafile/5G/Molecularmarker.tif]

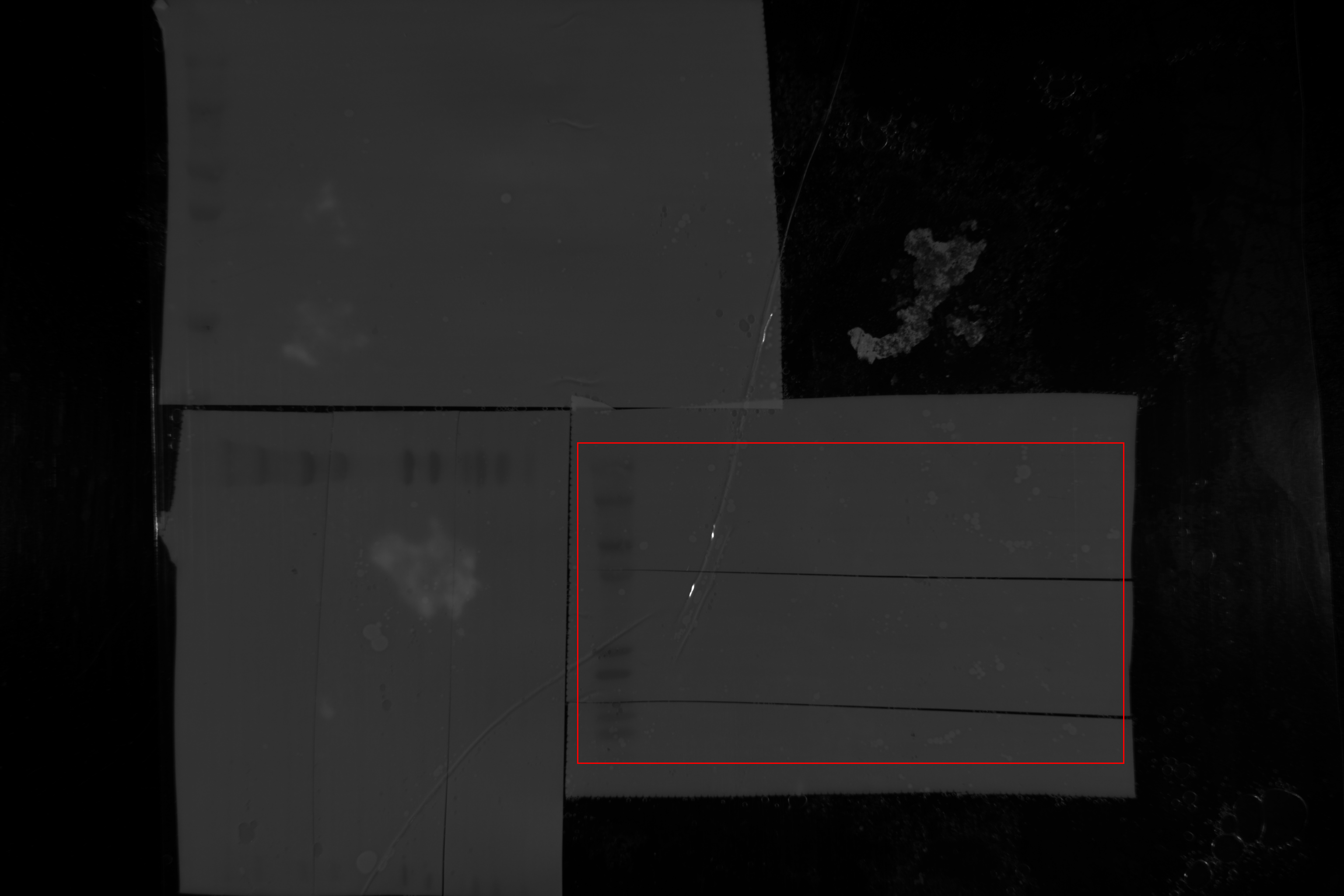

Supplement: Supplementary file 10 — Source data Fig. 6 [file 44318_2024_225_MOESM10_ESM.zip › Figure_6_sourcedatafile/6D/Molecularmarker.tif]

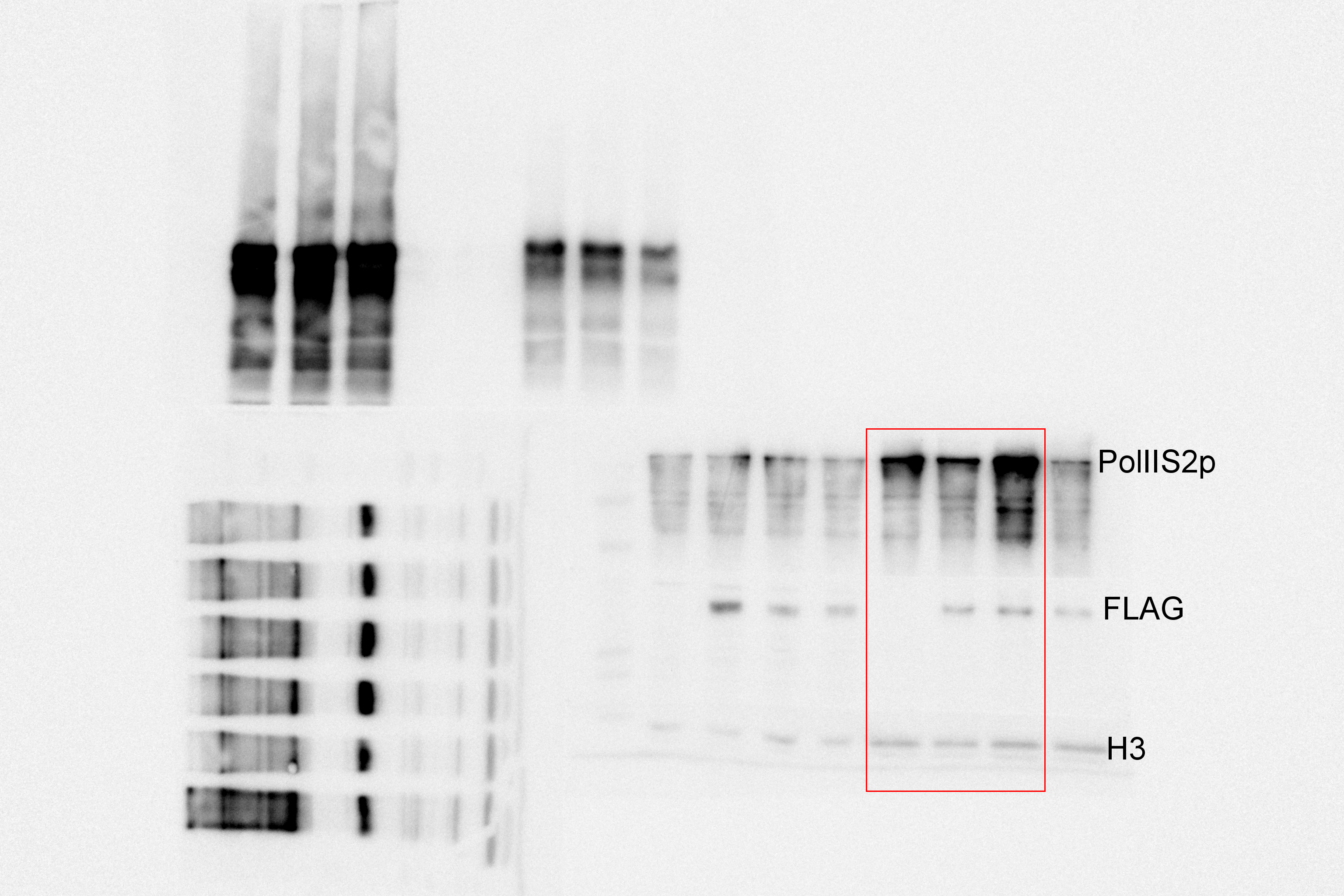

Supplement: Supplementary file 10 — Source data Fig. 6 [file 44318_2024_225_MOESM10_ESM.zip › Figure_6_sourcedatafile/6D/PolIIS2p_FLAG_H3WB.tif]
