# Supplementary material for: RNA fine-tunes estrogen receptor-alpha binding on low-affinity DNA motifs for transcriptional regulation
Source: EMBO J. 2024 Sep 16;43(21):5186–210. doi: 10.1038/s44318-024-00225-y (PMC11535219; doi:10.1038/s44318-024-00225-y)
Supplement: Supplementary file 11 — Expanded View Figures [file 44318_2024_225_MOESM11_ESM.pdf]

Expanded View Figures

Figure EV1. ERα interacts with RNA.

(**A**) Genome browser screenshot showing fRIP-seq IP, Input, and Log2F.C. (IP/Input) for MYC locus. (**B**) Genomic distribution of fRIP-seq peaks differentially enriched either in nucleoplasmic (NP) or chromatin (ca) fraction or distributed equally between chromatin and nucleoplasmic fractions. (**C**) fRIP-PCR using TFF1 eRNA oligos. (**D**) Immunoblot for ERα and GAPDH on RNA pulldowns using biotin-labeled TFF1 eRNA with lysates from cells grown in stripping media treated with either Vehicle or E2. (**E**) Summary plot showing the log2F.C. (fRIP IP/Input) across categories of ERα interacting RNA in Veh and E2 treatment. (**F**) Heatmap depicting ERα intensity on intergenic regions intersecting within 10 kb of caRNA or NPRNA or ca=NPRNA enriched fRIP-seq peaks. (**G**) Plot depicting the distance between the nearest ERα peak and fRIP-seq peak enriched either in caRNA (200 regions), NPRNA (275 regions) or ca=NP (583 regions) RNA. Statistical significance determined by Mann–Whitney *U*-test. Replicates for fRIP-seq and publicly available ERα ChIP-seq are mentioned in Appendix Table S6. (**H**) Illustration explaining the RNA-mediated recruitment of ERα.

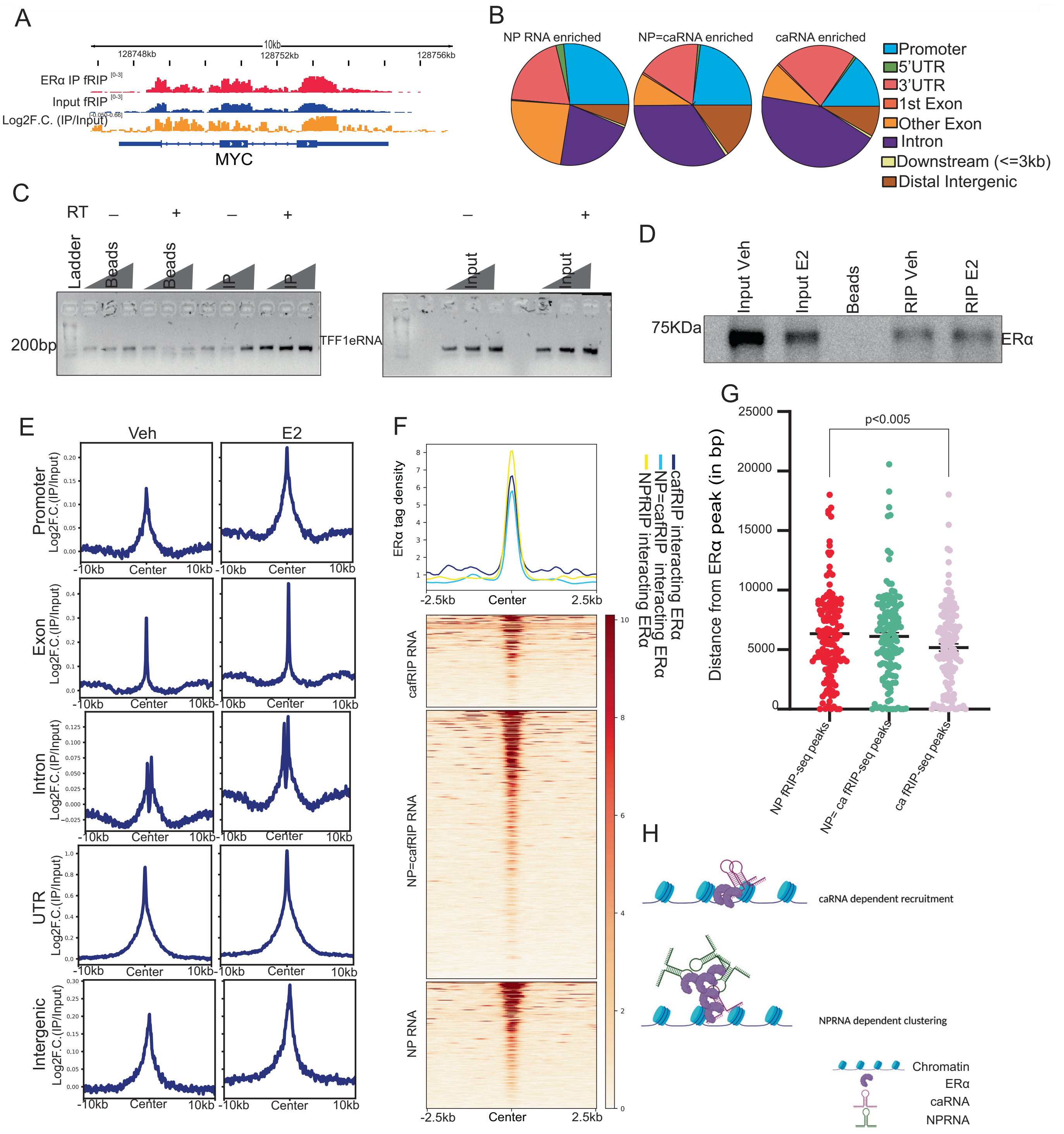
A
10kb
128748kb
128752kb
128756kb
ERα IP fRIP
Input fRIP
Log2F.C. (IP/Input)
MYC
B
NP RNA enriched
NP=caRNA enriched
caRNA enriched
Promoter
5'UTR
3'UTR
1st Exon
Other Exon
Intron
Downstream (<=3kb)
Distal Intergenic
C
RT
Ladder
Beads
IP
Input
200bp
TFF1eRNA
D
Input Veh
Input E2
Beads
RIP Veh
RIP E2
75KDa
ERα
E
Veh
E2
Promoter
Exon
Intron
UTR
Intergenic
Log2F.C.(IP/Input)
-10kb
Center
10kb
F
ERα tag density
-2.5kb
Center
2.5kb
cafRIP interacting ERα
NP=cafRIP interacting ERα
NPfRIP interacting ERα
cafRIP RNA
NP=cafRIP RNA
NP RNA
G
Distance from ERα peak (in bp)
p<0.005
NP fRIP-seq peaks
NP= ca fRIP-seq peaks
ca fRIP-seq peaks
H
caRNA dependent recruitment
NPRNA dependent clustering
Chromatin
ERα
caRNA
NPRNA

 © The Author(s)

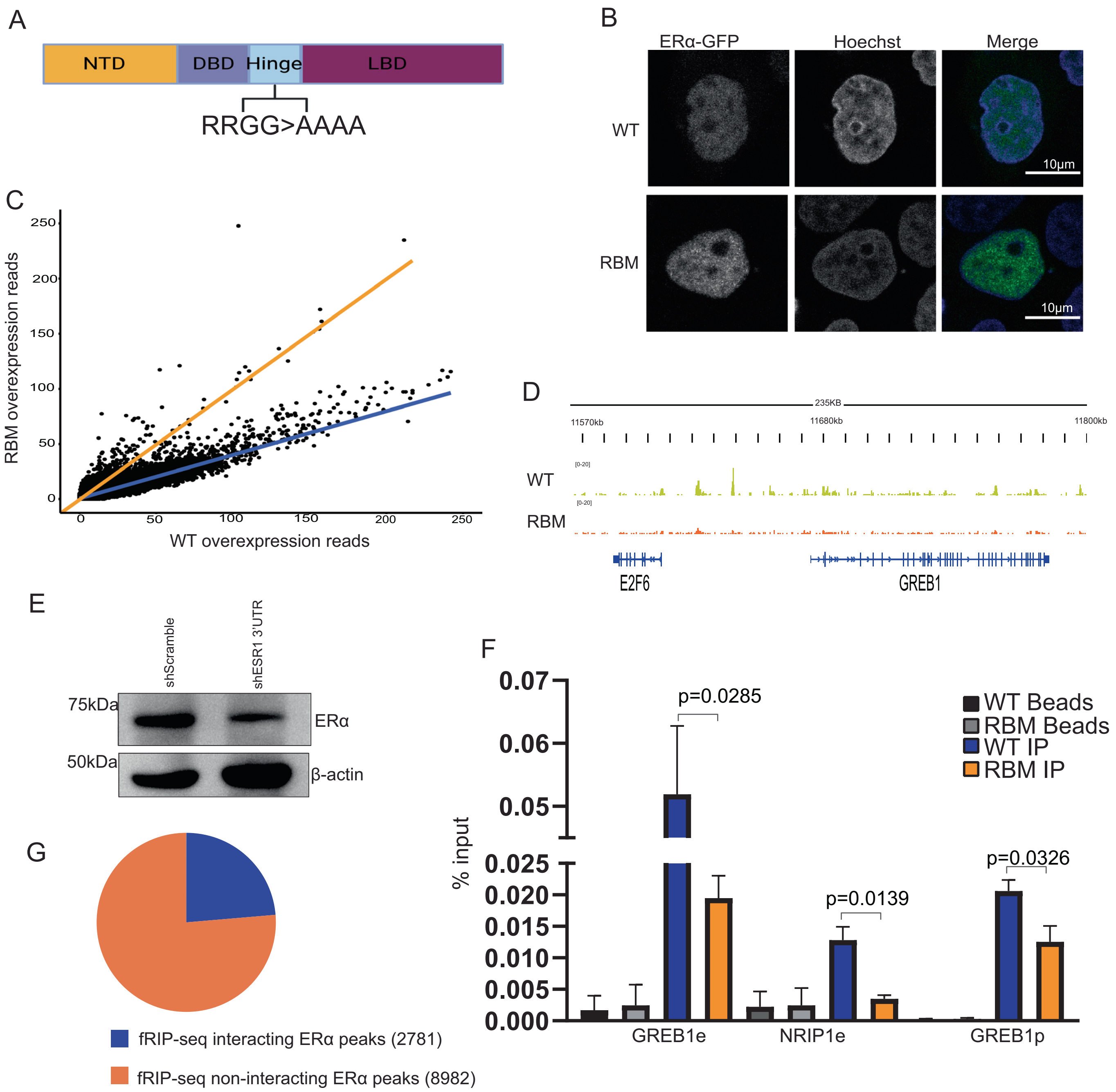

Figure EV2. RNA binding mutant of ERα shows loss of binding genome-wide.

(**A**) Schematic depicting the domains of ERα protein, the RNA binding RRGG sequence, and its mutation to AAAA. (**B**) Confocal images of ERα:GFP WT and RBM overexpressed in MCF-7 with E2 treatment for 1 h. (**C**) Normalized read counts showing the distribution of ERα:FLAG WT and RBM ChIP-seq reads. (**D**) Genome browser snapshot showing ChIP-seq signal of ERα WT and RBM on GREB1 locus in MCF-7. (**E**) Immunoblot for ERα and β-actin expression in total lysates from MCF-7 cells transfected with short hairpin RNA targeting against either a scramble sequence or the 3′UTR of the ESR1 gene. (**F**) FLAG enrichment on enhancers of GREB1 and NRIP1, as well as on the promoter of GREB1, in the background of downregulation of endogenous ERα and upon overexpression of WT and RBM FLAG-tagged ERα. Statistical significance was determined by unpaired *t*-test, and error bars denote the standard error of the mean (SEM) with two biological and three technical replicates. (**G**) Pie chart illustrating the number of ERα peaks categorized based on their interaction with RNA.

© The Author(s)

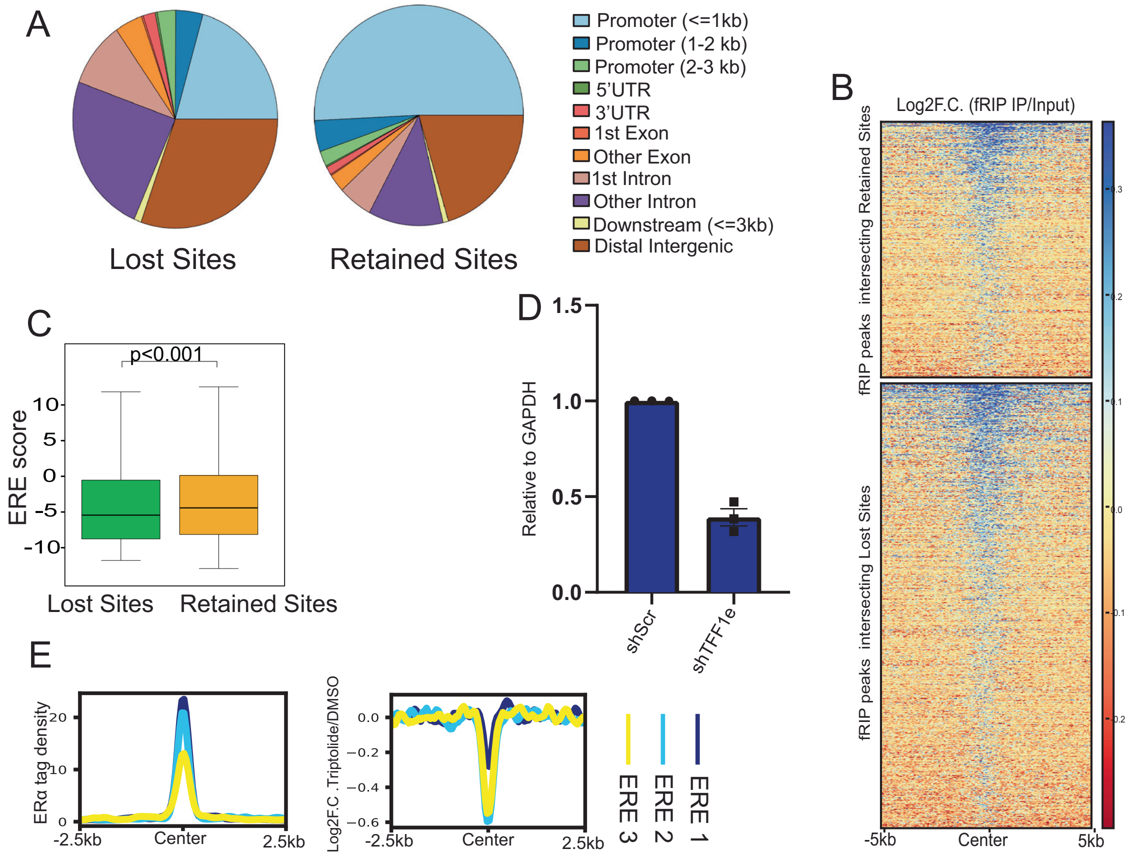

Figure EV3. Weaker motifs exhibit higher dependence on RNA for ERα binding.

(**A**) Genomic distribution of lost and retained peaks upon overexpression of RBM-ERα over WT-ERα. (**B**) Log2F.C. IP/Input for the fRIP-seq peaks intersecting with the lost and retained ERα upon RBM-ERα overexpression over WT-ERα. (**C**) Boxplot depicting the ERE motif score of ERα peaks that are lost (36,587 peaks) and retained (14,668 peaks) upon RBM expression as compared to the WT. Statistical significance determined by Mann–Whitney *U*-test. (**D**) qRT-PCR depicts the levels of TFF1 enhancer RNA following shRNA-mediated knockdown compared to scramble. The error bar denotes SEM from three biological replicates. (**E**) Heatmap depicting ERα tag density and Log2F.C (Triptolide ERα/DMSO ERα) tag density at varying ERE strength. The center lines of the boxplot denote the median, the box limits indicate the 25th and 75th percentiles, and the whiskers extend 1.5 times the interquartile range from the 25th and 75th percentiles. Outliers are not presented.

 © The Author(s)

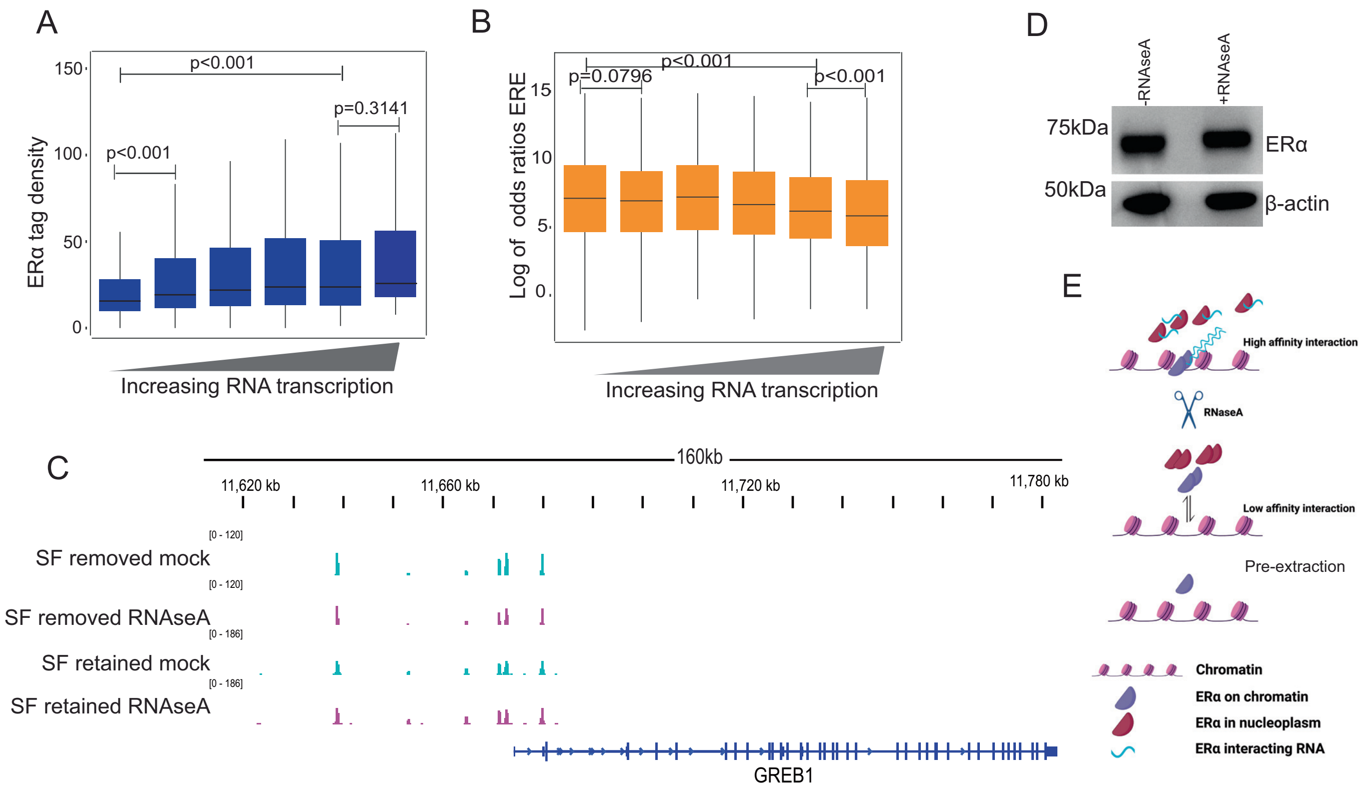

Figure EV4. ERα retention on chromatin is RNA dependent.

(**A**) Boxplot showing ERα enrichment on all sites binned based on the levels of RNA transcription in increasing order. Statistical significance determined by Mann–Whitney *U*-test. (**B**) Boxplot showing the Log of odds ratio for the ERE motif on all sites binned based on the levels of RNA transcription in increasing order. Statistical significance determined by Mann–Whitney *U*-test. (**C**) Genome Browser screenshot of GREB1 locus showing ERα ChIP-seq upon RNAse A treatment with removal and retention of soluble proteins. (**D**) Immunoblot depicting ERα and β-actin levels in MCF-7 isolated nuclei treated with mock or RNAse A. (**E**) Illustration depicting the RNA is required for ERα on chromatin. The center lines of the boxplot denote the median, the box limits indicate the 25th and 75th percentiles, and the whiskers extend 1.5 times the interquartile range from the 25th and 75th percentiles. Outliers are not presented. In (**A**, **B**), the first to sixth bins contains 15,863, 1404, 1988, 1543, 630, and 301 regions, respectively. Replicates for (**A**, **B**), publicly available ERα ChIP-seq, and GRO-seq are mentioned in Appendix Table S6.

© The Author(s)

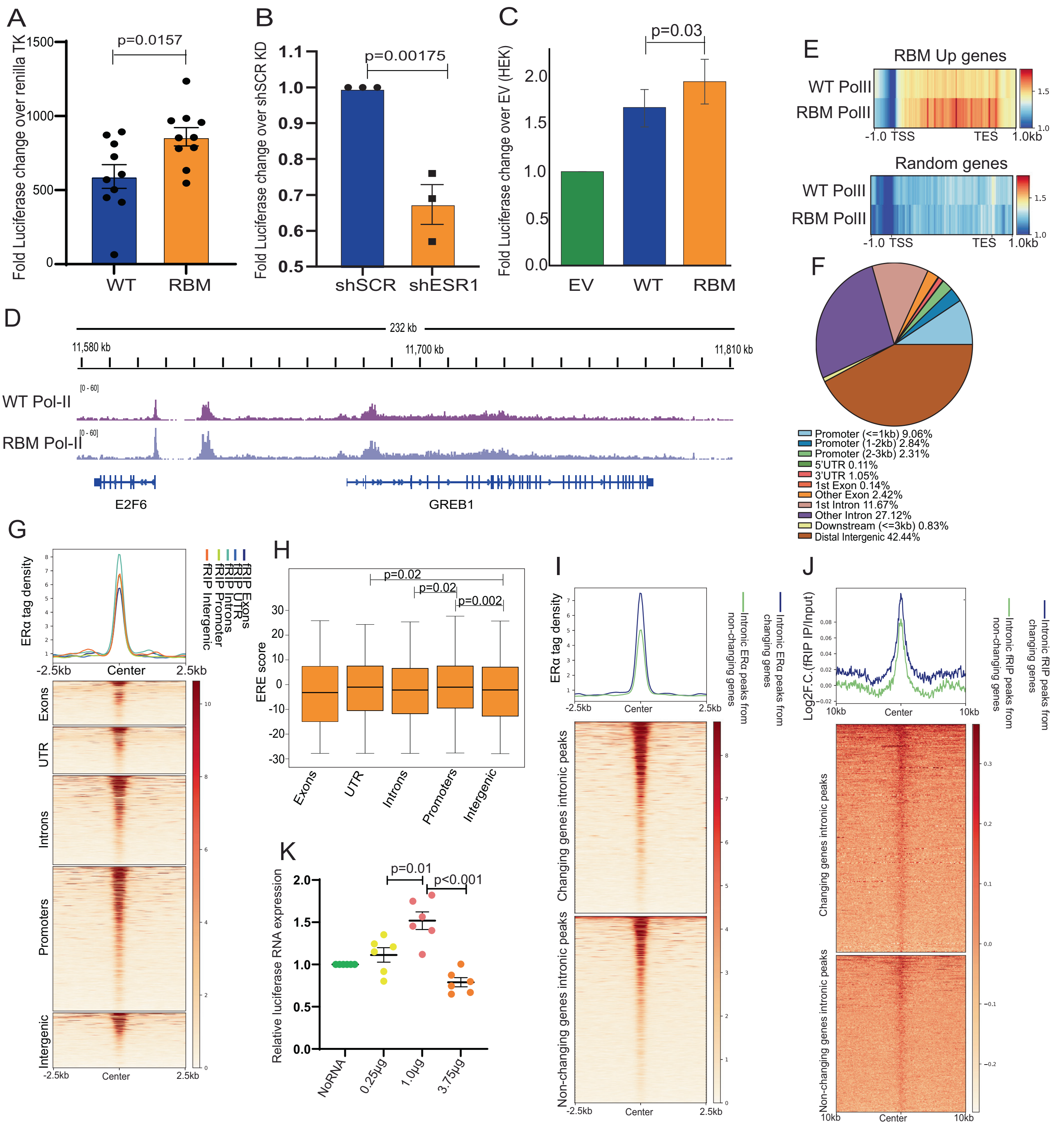
A
Fold Luciferase change over renilla TK
p=0.0157
WT
RBM
B
Fold Luciferase change over shSCR KD
p=0.00175
shSCR
shESR1
C
Fold Luciferase change over EV (HEK)
p=0.03
EV
WT
RBM
E
RBM Up genes
WT PolII
RBM PolII
-1.0 TSS
TES
1.0kb
Random genes
F
Promoter (<=1kb) 9.06%
Promoter (1-2kb) 2.84%
Promoter (2-3kb) 2.31%
5'UTR 0.11%
3'UTR 1.05%
1st Exon 0.14%
Other Exon 2.42%
1st Intron 11.67%
Other Intron 27.12%
Downstream (<=3kb) 0.83%
Distal Intergenic 42.44%
D
232 kb
11,580 kb
11,700 kb
11,810 kb
WT Pol-II
RBM Pol-II
E2F6
GREB1
G
ERα tag density
fRIP Exons
fRIP UTR
fRIP Introns
fRIP Promoter
fRIP Intergenic
-2.5kb
Center
2.5kb
Exons
UTR
Introns
Promoters
Intergenic
H
ERE score
p=0.02
p=0.02
p=0.002
Exons
UTR
Introns
Promoters
Intergenic
K
Relative luciferase RNA expression
p=0.01
p<0.001
NoRNA
0.25μg
1.0μg
3.75μg
I
ERα tag density
Intronic ERα peaks from changing genes
Intronic ERα peaks from non-changing genes
Changing genes intronic peaks
Non-changing genes intronic peaks
J
Log2F.C.(fRIP IP/Input)
Intronic fRIP peaks from changing genes
Intronic fRIP peaks from non-changing genes
10kb
Center
10kb

© The Author(s)

Figure EV5. The dynamic binding of ERα allows better transcription of target genes.

(**A**) 3X ERE-driven firefly luciferase activity normalized to renilla TK luciferase upon 24 h of E2 treatment. Statistical significance determined by Mann–Whitney *U*-test and error bar denotes SEM from five biological replicates with two technical replicates each, plotted all. (**B**) Luciferase activity normalized to the scramble knockdown for the 3X ERE reporter assay conducted with short hairpin RNA targeting either the scramble sequence or the 3′UTR of the ESR1 gene in MCF-7 cells after 24 h of E2 treatment. Statistical significance was determined using the Mann–Whitney *U*-test, and the error bar denotes SEM from three biological replicates. (**C**) 3X ERE-driven firefly luciferase activity upon WT-ERα or RBM-ERα overexpression and 24 h of E2 treatment in HEK-293T. Statistical significance determined by Mann–Whitney *U*-test and error bar denotes SEM from three biological replicates. (**D**) Genome browser screenshot showing the occupancy of total PolII on GREB1 locus upon expression of WT-ERα or RBM-ERα. (**E**) Profile plot illustrating PolII tag density normalized with respect to *Drosophila* DNA spike in, plotted on genes upregulated by RBM and a random set of genes across the entire gene body. (**F**) Pie chart depicting the genomic distribution of ERα ChIP-seq peak. (**G**) Heatmap depicting the ERα intensity across ERα bound regions within 10 kb of various categories of fRIP-seq peaks. (**H**) Boxplot depicting the ERE motif score from different categories of exonic (445), UTR (486), intronic (943), promoter (1574), and intergenic (574), ERα peaks interacting with RNA. Statistical significance was determined using the Mann–Whitney *U*-test. (**I**) Heatmap depicting the ERα tag density on intronic sites from changing and non-changing gene categories. (**J**) Heatmap depicting the fRIP-seq signal from intronic peaks of genes that are changing and non-changing. (**K**) Relative luciferase RNA expression from an in vitro transcription reaction with varying concentrations of added RNA. Statistical significance is determined by unpaired *t*-test and error bar denotes SEM from three biological replicates. The center lines of the boxplot denote the median, the box limits indicate the 25th and 75th percentiles, and the whiskers extend 1.5 times the interquartile range from the 25th and 75th percentiles. Outliers are not presented.
